# Horizontal gene transfer and the evolution of transcriptional regulation in *Escherichia coli*

**DOI:** 10.1186/gb-2008-9-1-r4

**Published:** 2008-01-07

**Authors:** Morgan N Price, Paramvir S Dehal, Adam P Arkin

**Affiliations:** 1 Physical Biosciences Division, Lawrence Berkeley National Laboratory, 1 Cyclotron Road, Mailstop 977-152, Berkeley, California 94720, USA; 2 Virtual Institute of Microbial Stress and Survival, Lawrence Berkeley National Laboratory, 1 Cyclotron Road, Mailstop 977-152, Berkeley, California 94720, USA; 3 Department of Bioengineering, 1 Cyclotron Road, Mailstop 977-152, University of California, Berkeley 94720, California, USA

## Abstract

**Background:**

Most bacterial genes were acquired by horizontal gene transfer from other bacteria instead of being inherited by continuous vertical descent from an ancient ancestor. To understand how the regulation of these acquired genes evolved, we examined the evolutionary histories of transcription factors and of regulatory interactions from the model bacterium *Escherichia coli *K12.

**Results:**

Although most transcription factors have paralogs, these usually arose by horizontal gene transfer rather than by duplication within the *E. coli *lineage, as previously believed. In general, most neighbor regulators - regulators that are adjacent to genes that they regulate - were acquired by horizontal gene transfer, whereas most global regulators evolved vertically within the γ-Proteobacteria. Neighbor regulators were often acquired together with the adjacent operon that they regulate, and so the proximity might be maintained by repeated transfers (like 'selfish operons'). Many of the as yet uncharacterized (putative) regulators have also been acquired together with adjacent genes, and so we predict that these are neighbor regulators as well. When we analyzed the histories of regulatory interactions, we found that the evolution of regulation by duplication was rare, and surprisingly, many of the regulatory interactions that are shared between paralogs result from convergent evolution. Another surprise was that horizontally transferred genes are more likely than other genes to be regulated by multiple regulators, and most of this complex regulation probably evolved after the transfer.

**Conclusion:**

Our findings highlight the rapid evolution of niche-specific gene regulation in bacteria.

## Abstract 2

Most Escherichia coli transcription factors have paralogs, but these usually arose by horizontal gene transfer rather than by duplication within the E. coli lineage, as previously believed.

## Background

Transcription factors (TFs) bind to specific sites on DNA where they regulate the expression of target genes and thus allow bacteria to adapt to a changing environment. In the well studied bacterium *Escherichia coli *K12, more than 150 TFs have been characterized [1] and nearly 100 more are predicted from the genome sequence. Most of the *E. coli *TFs include a DNA-binding domain that determines target site specificity as well as a sensing domain that binds to small metabolites or to signaling proteins [2]. With the availability of complete genome sequences from diverse bacteria, researchers have begun to consider how these TFs and their binding sites evolved [2-6].

### Evolution of regulation by duplication?

Because *E. coli *TFs form large families of homologous proteins, the interpretation has been that most of them arose by gene duplication [2,7]. Two TFs from any given family usually regulate distinct genes and bind to distinct effectors; the duplicates therefore generally have distinct rather than overlapping functions. However, it has not been clear from previous studies whether the duplicates arose within the *E. coli *lineage [8] or were acquired by horizontal gene transfer (HGT), or how long ago these duplication events occurred. For example, the ancestral TF might have been transferred to another lineage, where it diverged and acquired a new function, and could then have been reacquired, to give paralogs that arose by HGT rather than by duplication within the *E. coli *lineage [9]. This is termed 'allopatric gene divergence'.

It has also been proposed that gene duplication is a major source of regulatory interactions. Although paralogous TFs usually have different functions, there are many cases in *E. coli *in which paralogous TFs regulate the same genes, or paralogous genes are regulated by the same TF, and a few cases where paralogous genes are regulated by paralogous TFs [4]. Between 7% [2] and 38% [4] of the regulation in *E. coli *is reported to have arisen by gene duplication, although another group reported that this is rare [7]. Also, about one-third of paralogous genes are reported to have conserved operon structure [10] and conserved regulatory sequences [3]. Because these studies did not examine whether the paralogs were closely related and whether the regulation was conserved from an ancestral state, these regulatory similarities could have evolved independently, instead of being conserved from the common ancestors of the genes.

### Evolution of regulatory sites

The evolution of the regulatory sites that TFs bind to has also been studied by comparing upstream sequences across *E. coli *and its relatives [3,11,12]. It appears that regulatory sites are usually conserved in close relatives within the family of Enterobacteria, such as *Salmonella typhimurium *and *Klebsiella pneumoniae*, and are often also conserved in moderately distant relatives within the γ-Proteobacterial division, such as *Vibrio cholerae *or *Shewanella oneidensis*. So, many of these regulatory sites are quite old [3,11,12]. This also implies that these regulatory sites are under strong purifying (negative) selection.

However, because these studies compared orthologous genes in *E. coli *and its relatives, they did not examine the regulation of recently acquired genes. As most of the genes in *E. coli *K12 were acquired by HGT after the divergence of the γ-Proteobacteria [13], it is important to consider how acquired genes are regulated. HGT genes may evolve new regulation after they are acquired, either because the genes' regulators from the source bacterium are not present in the new host or because different conditions in the new host select for different regulation. On the other hand, newly acquired genes might be more likely to be fixed in the population if they already contain regulatory sequences that can function in their new host. Thus, the evolutionary origin of the regulation of acquired genes also has broader implications for our understanding of HGT.

### Neighbor regulators evolve by HGT?

Finally, it has been observed that many of the regulators in *E. coli *are adjacent to operons that they regulate [14]. These 'neighbor regulators' usually regulate just one or two operons, and the proximity of these regulators to their regulated genes suggests that HGT might be involved in the evolution of these regulatory relationships [14]. Furthermore, these neighbor regulators are often conserved adjacent to their targets in other genomes [15]. However, as far as we know, there has not been a direct test of whether neighbor regulation is associated with HGT.

### Evolutionary histories of TFs

To clarify the origins of transcriptional regulation in *E. coli*, we conducted a detailed phylogenetic analysis of its TFs. This allowed us to distinguish paralogs that have been maintained in the lineage since their duplication from paralogs that were acquired by HGT. We found that relatively few of the TFs evolved by duplications within the *E. coli *lineage. Instead, we found a surprisingly complex history of HGT for many of the regulators, especially for the neighbor regulators and the as yet uncharacterized regulators. Furthermore, these specific regulators are often co-transferred together with their regulated genes, which allows us to predict regulatory targets. In contrast, most of the global regulators appear to have ancient origins in the γ-Proteobacteria.

### Convergent evolution of regulatory interactions

We then analyzed the histories of individual regulatory interactions. To determine whether gene regulation evolves by duplication, we examined the evolutionary histories of regulatory interactions that are shared between paralogs in one of the three ways listed above (paralogous TFs that regulate the same gene, paralogous genes that are regulated by the same TF, or paralogous genes that are regulated by paralogous TFs). Specifically, we compared the age of these shared regulatory interactions with the age of the duplication that created the paralogs. To date each regulatory interaction, we assumed that the interaction is no older than the presence of both TF and regulated gene in the *E. coli *lineage. We found that the regulatory similarities between paralogs usually evolved after the duplication event, rather than being conserved from their common ancestor, as has been assumed [4]. This shows that little of the regulatory network was created by duplication.

Furthermore, these similarities between paralogs are much more common than expected by chance. It appears that gene regulation is subject to convergent evolution, and so related genes independently evolve regulatory interactions with the same (or similar) genes. Although convergent evolution at the molecular level is usually thought of in terms of protein function, here the key functional features are the genes' upstream regulatory regions, which independently (and hence convergently) evolve to bind the same regulators or to bind related regulators. Of course, many TFs bind upstream of multiple genes, and in most cases those binding sites also evolved independently. We use the term 'convergent evolution' for paralogs to emphasize that their binding sites evolved independently, and not by duplication.

### Regulation of acquired genes

**Figure 1:**
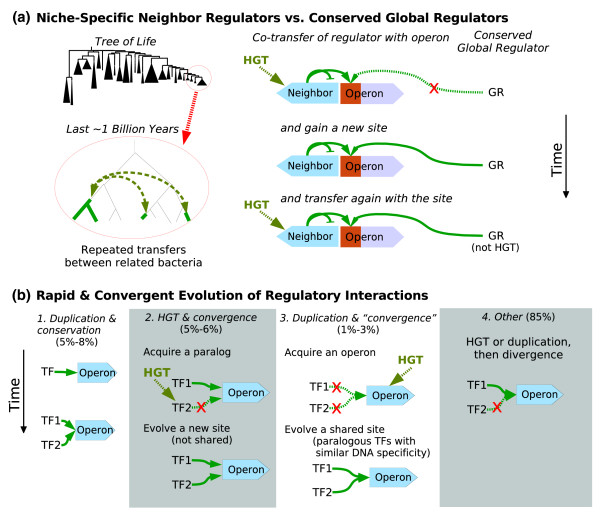
Evolutionary history of regulators and regulatory interactions. **(a) **Most of the transcription factors (TFs) regulate adjacent genes. These 'neighbor regulators' are often transferred between related bacteria and are often lost, and so they seem to be niche specific. Neighbor regulated genes are often regulated by other regulators as well, but this regulation is usually not conserved across horizontal gene transfer (HGT) events. **(b) **Scenarios for the evolution of regulatory interactions. For each scenario, we show the proportion of known regulatory interactions in *E. coli *[1] that evolved that way. Scenario 1: regulatory interactions are conserved after gene duplication in a small fraction of cases. Scenario 2: even when paralogous TFs or paralogous regulated genes have similar regulatory interactions, this often results from the evolution of similar regulation after HGT, rather than being conserved from the duplication event. Scenario 3: in some cases, a single region of DNA evolves to bind two paralogous TFs. Unlike scenario 2, this scenario relies on the similarity of the TFs. Scenario 4: Most TFs, and probably most other genes as well, ultimately arose by a duplication, either within a lineage or by allopatric gene divergence. Nevertheless, the regulatory interactions are usually not shared with their paralogs. (To estimate a frequency for scenario 4, we assumed that all genes arose by some kind of duplication.) Separate results for paralogous TFs, for paralogous regulated genes, and for paralogs of both are given in Table 1.

Because global regulators are strongly conserved and account for more than half of all known regulatory interactions [1], we wondered how they relate to HGT genes. We found that HGT genes tend to be under more complex regulation than native genes, and the global regulator CRP regulates a higher proportion of HGT genes than of native genes. We identified cases in which regulatory sites for conserved global regulators have been conserved across HGT events within the γ-Proteobacteria, but most of the regulation of these HGT genes appears to have evolved after the transfer event. This illustrates that major parts of the regulatory network evolved recently under selection. Overall, most of the TFs have been acquired recently and, even for the global regulators, most of the binding sites have evolved relatively recently. We provide a schematic overview of our results in Figure 1.

## Results and discussion

### Evolutionary histories of transcription factors

**Figure 2:**
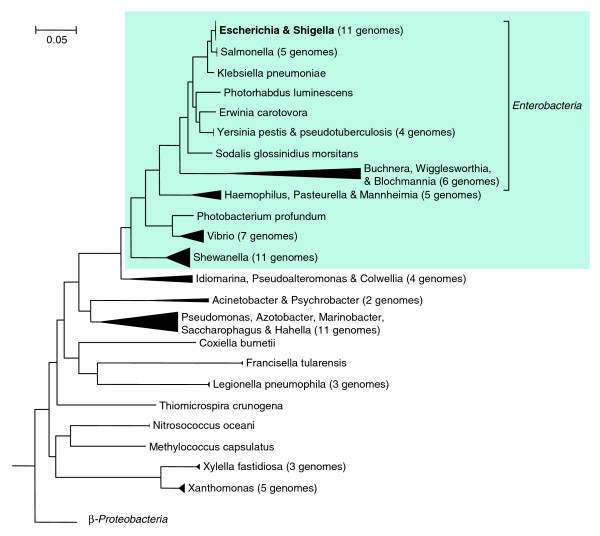
Phylogeny of the γ-Proteobacteria. The phylogeny was derived from concatenated alignments of highly conserved proteins (see Materials and methods). In this study, we focused on evolutionary events after the divergence of *Shewanella *spp. from *Escherichia coli *K12 (the shaded portion of the tree). The β-Proteobacteria formed a sister group to the γ-Proteobacteria. The scale bar corresponds to 5% amino acid divergence.

Because most TFs belong to large families and have paralogs, we built phylogenetic trees for the TFs (see Materials and methods, below) and we manually compared these trees with the species tree shown in Figure 2. We focused on the period after the divergence of *E. coli *from *Shewanella*, because we found phylogenetic reconstruction deeper within the γ-Proteobacteria to be impractical. (Most gene trees are poorly resolved beyond this distance, probably because the phylogenetic signal is reduced once the sequence divergence becomes too great.) According to our species tree (see Materials and methods, below), this period comprises about a third of *E. coli*'s evolutionary history since the divergence of the bacteria, or perhaps 1 billion years. As we see below, much as changed during this time.

**Figure 3:**
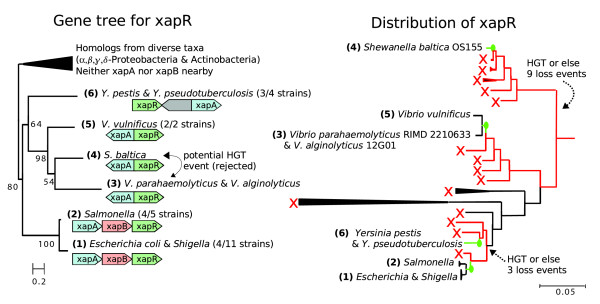
Repeated co-transfer of *xapR *with *xapA*, which it regulates. In the presence of xanthosine, *xapR *activates the transcription of the *xapAB *operon, which allows the transport and catabolism of xanthosine [65]. The gene tree shows that *xapR *forms a well supported clade (80/100 bootstraps) within a larger family of regulators (COG583). *xapR *is scattered across the γ-Proteobacteria, within which we identify four acquisition events. For each acquisition, we show the multiple independent gene losses that would otherwise be required to explain the gene's distribution across the species tree. The gene tree also places *xapR *from *Shewanella baltica *between the sequences from *Vibrio *spp., which suggests that it could have been acquired separately by the two groups of *Vibrio*. However, this potential fifth acquisition event is rejected because of several factors: the bootstrap support is low; a small change to the tree's topology (one swap) would render the gene tree congruent with the species tree; and the gene might have been transferred from an ancestor of one of these *Vibrio *spp. to *S. baltica*. The *xapR *tree was computed from amino acid sequences using phyml with 100 bootstraps, four classes of gamma-distributed rates (with optimized alpha), and an optimized proportion of invariant sites [55]. In the gene tree, the scale bar corresponds to 20% amino acid divergence, and the internal nodes are labeled with their bootstrap values. The gene context shows gene order only (not spacing or scale).

**Figure 4:**
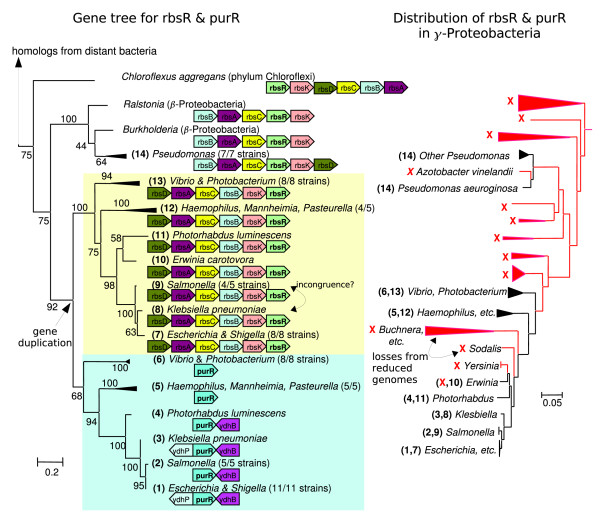
The regulator *purR *evolved by duplication from the ribose repressor *rbsR*, itself acquired by HGT. Within the Enterobacteria/Vibrionaceae subgroup of the γ-Proteobacteria, both *rbsR *and *purR *exhibit largely vertical evolution. The closest relatives of *rbsR *and *purR *from outside this subgroup of γ-Proteobacteria are associated with genes for ribose utilization and probably function as ribose repressors. The absence of both *rbsR *and *purR *from *Buchnera *and its relatives and from *Sodalis *might suggest additional transfer events, but because *Buchnera *and its relatives have under 700 genes, absence from this clade is not evidence for horizontal gene transfer (HGT). *Sodalis *is also a reduced genome, with around 2,600 genes, whereas most Enterobacteria have over 4,000 genes. The *purR*/*rbsR *tree was computed from protein sequences with phyml and 100 bootstraps (as in Figure 3).

**Figure 5:**
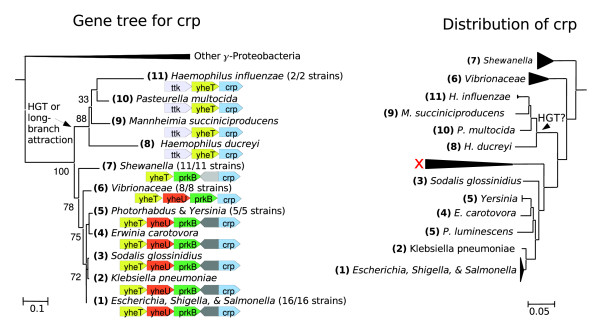
The global regulator *crp *has undergone predominantly vertical evolution. *Crp *has conserved context, and the gene tree is concordant with the species tree except for the Pasteurellacea and perhaps *Sodalis*. The incongruent placement of *Sodalis *is not supported by a nucleotide sequence tree (data not shown). The deep branching of the Pasteurellacea is strongly supported, and two swaps would be required to make its placement concordant with the species tree. An insertion of *crp *into Pasteurellacea is unlikely because of the conserved proximity of the functionally unrelated gene *yheT*. Instead, the placement probably reflects homologous recombination or long branch attraction. In any case, this does not affect the lineage leading to *Escherichia coli*, and so we classified *crp *as native. The *crp *tree shown was computed from protein sequences with phyml and 100 bootstraps (as in Figure 3).

We classified a TF as being acquired by HGT after this divergence if close relatives of the TF were found in more distantly related bacteria, so that three or more gene loss events would otherwise be required to reconcile the gene tree with the species tree (for example, see Figure 3; see Materials and methods, below, for details). We classified a TF as being duplicated within the *E. coli *lineage if it had a paralog that was closely related in the gene tree (for example, Figure 4). We classified a gene as an 'ORFan' if it had no homologs in organisms more distantly related than *Shewanella*. The origin of microbial ORFans is unclear [16], but they might be HGT from an unknown source. Finally, we classified other TFs as native (evolving by vertical descent; for example, Figure 5). However, because our criteria for identifying HGT was conservative, there may be undetected HGT events within the 'native' TFs, as well as ancient HGT before the divergence of *E. coli *from *Shewanella*.

**Figure 6:**
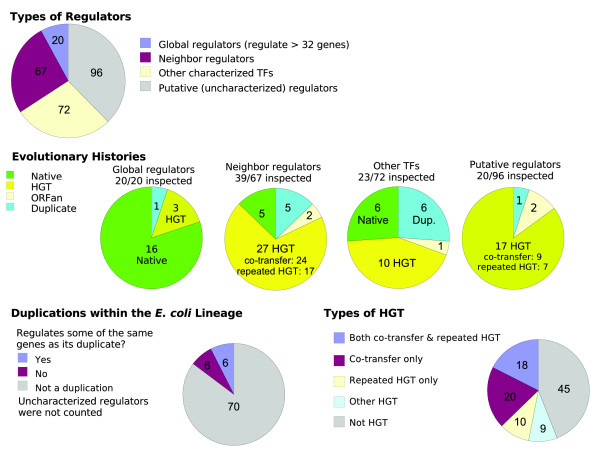
Evolutionary histories of *Escherichia coli *TFs. We classified characterized regulators as global regulators, neighbor regulators, or other regulators, and we also analyzed some putative (as yet uncharacterized) regulators. We classified these transcription factors (TFs) as native because the divergence of *E. coli *from *Shewanella*, as acquired by horizontal transfer after that divergence, as ORFan (indicating horizontal gene transfer [HGT] from an unknown source), or as duplications within the *E. coli *lineage. For the duplicated TFs, we examined whether they regulate the same genes as their duplicates. For the HGT regulators, we examined whether they were co-transferred with nearby genes and whether they underwent repeated HGT within γ-Proteobacteria.

Besides phylogeny, we also classified TFs by their function. We analyzed characterized transcription factors from RegulonDB 5.6 [1]. We classified the 20 TFs that regulated the largest number of genes as global regulators. We classified TFs that regulate adjacent genes as neighbor regulators. To exclude autoregulation, which is common, we classified TFs as neighbor regulators only if they regulate adjacent yet distinct transcription units. (Five of the global regulators also regulate adjacent operons; those were excluded from the neighbor regulators.) We also considered other characterized TFs and putative, as yet uncharacterized regulators. We analyzed the history of each of the global regulators, and of a sample of each of the other types of regulators (see Figure 6 and Materials and methods, below; for data on individual TFs, see Additional data file 1).

Whereas most global regulators were native genes within the γ-Proteobacteria, most neighbor regulators have been acquired after the divergence of the *E. coli *and *Shewanella *lineages (Figure 6). Other characterized regulators were native, HGT, or duplications within the lineage leading to *E. coli*, in roughly equal proportions. Finally, most of the putative regulators were acquired by HGT (Figure 6). Overall, we found little duplication of TFs within the *E. coli *lineage. In the following sections we examine in more detail the global regulators, the neighbor regulators, and the pattern of HGT.

#### Vertical evolution of most global regulators

We found that 17 out of the 20 global regulators have evolved vertically since the divergence of *E. coli *from *Shewanella*. For example, as shown in Figure 5, *crp *has mostly evolved vertically, with no evidence for gene gain and with gene losses only in the highly reduced genomes of the insect endosymbionts. There may have been homologous recombination, however.

Our finding that global regulators are gained and lost more slowly than other regulators complements a report that global regulators, as defined by their weak DNA binding specificity, undergo slower sequence evolution than other regulators [3]. However, the previous report used bidirectional best Basic Local Alignment Search Tool (BLAST) hits to identify orthologous TFs, which can give misleading results [17]. To confirm that the sequence of global regulators evolves slowly, we examined 40 evolutionary orthologs of characterized TFs between *E. coli *and *Shewanella oneidensis *MR-1. These orthologs were identified by an automated analysis of phylogenetic trees [18] and were confirmed by inspection. We found a clear correlation between conservation (defined as the BLAST bit score divided by the self score for the *E. coli *gene) and the number of genes that the TF is reported to regulate in RegulonDB (Spearman ρ = 0.48, *P *< 0.002, *n *= 40; see Additional data file 2). Thus, global regulators do evolve more slowly than other regulators, both in terms of gene gain and gene loss and in their amino acid sequence.

#### Co-transfer of neighbor regulators with regulated genes

In contrast to global regulators, most neighbor regulators were acquired by horizontal transfer. Neighbor regulators were also marginally more likely than other non-global regulators to be HGT (*P *= 0.06, by Fisher's exact test). To determine whether these neighbor regulators were co-transferred with nearby genes that they regulate, we considered whether the TF and regulated gene(s) had xenologs that were near each other. (Xenologs are homologs that are related to each other by HGT rather than by vertical descent.) Of the 39 neighbor regulators that we inspected, 27 were classified as HGT, and 24 of those have been acquired by co-transfer with one or more of their regulated genes (for example, *xapR *with *xapA *in Figure 3). In contrast, a previous analysis [5] revealed that bacterial TFs do not usually co-evolve with their regulated genes. The previous analysis relied on bidirectional best BLAST hits, and for TFs these hits are often spurious [17].

It has also been proposed that repressors are more likely than activators to co-evolve with their regulated genes [19]. However, we found that activators, repressors, and dual regulators were equally likely to be co-transferred with their regulated genes (see Additional data file 1). The discrepancy might arise because we looked for co-transfer events, whereas the previous work looked for gene loss events. In other words, the regulators are co-evolving with their genes by HGT, regardless of the sign of the regulation, but activators are more likely to be lost, perhaps as the first step toward loss of the entire pathway [19]. Indeed, both of the regulators whose loss is discussed in detail in the previous work have undergone co-transfer with regulated genes (*flhDC *with *fliA *and *fliD*, and *malT *with *malS*; see Additional data file 1). Overall, HGT appears to be associated with neighbor regulation, and a majority of neighbor regulators have been co-transferred with their regulated genes.

#### Most uncharacterized regulators are neighbor regulators

We considered that co-transfer might be used to predict the function of uncharacterized regulators. To determine whether such predictions would be reliable, we looked for co-transfer events among the 38 non-neighbor regulators (including global regulators) that we examined. We also looked for co-transfer events involving TFs that are known [1] or predicted [20] to be in operons. We found ten additional co-transfer events, and in seven of these cases the co-transferred genes are regulated by the TF. (In most of these cases the TF was not classified as a neighbor regulator because it was co-transcribed with the regulated genes.) The three exceptions were as follows: *fecR *has been co-transferred with its sensor *fecI*; *alpA *has been co-transferred with *yfjI *as part of prophage CP4-57 [21]; and the flagellar regulator *flhDC *has co-transferred with *motAB*, which is also involved in chemotaxis. Overall, co-transfer was not a 100% reliable indicator of regulation, but we found few exceptions relative to the large number of co-transfer events that did indicate regulation (3 versus 30), and in all cases the co-transferred genes did have related functions.

We then analyzed, by hand, the evolutionary history of a random sample of 20 uncharacterized regulators. (We chose genes that contain a putative DNA-binding domain but are neither characterized nor annotated with another function [see Materials and methods, below].) We found that most of these uncharacterized regulators were acquired by HGT (17/20; Figure 6). Almost half of them (9/20) were co-transferred with adjacent genes. This proportion is similar to the proportion of neighbor regulators that are co-transferred (24/39). (The proportions are not significantly different [*P *> 0.2, by Fisher's exact test].) Hence, we predict that most of the as yet uncharacterized regulators in *E. coli *are neighbor regulators. We also predict that most of the uncharacterized regulators control the expression of just one or two operons, as is seen for the characterized neighbor regulators [14].

We tried to identify co-transfer automatically by searching for conserved proximity in distant organisms, but without much success. We used bidirectional best hits to identify potential orthologs in those organisms, and although these best hits are often false positives we hypothesized that testing for conserved proximity would eliminate the false positives. Unfortunately, this automated approach did not identify most of the co-transferred TFs that we identified manually (data not shown). Many of the HGT events are between *E. coli *and related bacteria (discussed below), and detailed phylogenetic analysis is required to uncover these HGT events. Conserved proximity has also been used in combination with orthology groups (clusters of orthologous groups of proteins [COGs] [22]) to identify regulatory relationships [15]. That study made many successful predictions but also had a high rate of false positives because of the difficulty in automatically placing TFs into orthology groups [15]. Thus, automating the identification of co-transfer is beyond the scope of this report.

#### Repeated HGT of regulators between related bacteria

While examining the neighbor regulators, we sometimes found that close homologs of these regulators had sporadic distributions in *E. coli *and its relatives (for example, *xapR *in Figure 3). We classified as 'repeated HGT' those genes whose sporadic distributions implied two or more HGT events within the γ-Proteobacteria. (As previously, we inferred an HGT event when three or more independent deletion events would otherwise be required to explain the distribution across species of a clade in the gene tree.) By this restrictive definition, we found repeated HGT between relatives for 17 of the 39 neighbor regulators that we examined, which indicates both a strong preference for gene transfer within γ-Proteobacteria and high rates of gene gain for this class of genes.

Previous studies have disagreed as to whether HGT of regulatory genes is relatively common [23] or relatively rare [24]. The study that found that HGT of regulatory genes was rare relied on clusters that contained only one gene per genome to define gene families [24]. Such clusters might be difficult to identify for large families such as TFs. Although we do not compare the rate of HGT for regulators with the rate of HGT for other types of genes, we find high rates of HGT for regulators, with the exception of a few global regulators (Figure 6).

Previous studies have also disagreed as to whether HGT within the γ-Proteobacteria is prevalent [24,25] or not [13,26]. To confirm that HGT between related bacteria is common, we used an automated procedure, based on the presence and absence of close homologs of a gene, to identify potential HGT events (see Materials and methods, below). We then considered whether the closest xenologs of these HGT genes were from related bacteria. We found that these closest xenologs were far more likely to be from related bacteria than expected by chance (*P *< 10^-15^, by binomial test; see Additional data file 3). Because identifying HGT between related genomes requires large numbers of genome sequences, so that the absence of the gene from intermediate genomes can be confirmed (for example, see Figure 3), too few genomes may have been available for previous studies to observe this trend. For example, we analyzed 87 γ-Proteobacterial genomes, whereas Lerat and coworkers [13] analyzed only 13 γ-Proteobacteria.

### Evolutionary histories of regulatory interactions

#### Little of gene regulation arises by duplication

As discussed above, most of the TFs that we analyzed appear to have arisen by HGT events rather than by duplications within the *E. coli *lineage. If we extrapolate from the TFs tabulated in Figure 6, and correct for the uneven sampling of different types of regulators, then 33 ± 7 of the 255 regulators in *E. coli *arose by lineage-specific duplications, and 163 ± 10 regulators were acquired by HGT. (We estimated these standard errors by simulating data according to the observed frequencies within each type of regulator [parametric bootstrap].) Thus, although bacterial TFs form large families that often have many representatives within a single genome, these representatives are largely xenologs that arose by HGT, rather than being evolutionary paralogs that arose by duplication within the *E. coli *lineage.

When we examined the few TFs that did arise by lineage-specific duplication, we found that many of them do not share regulation with their paralogs. We must exclude uncharacterized TFs, and we also excluded autoregulation, which is reported for over half of the characterized TFs in RegulonDB and which need not be conserved from the common ancestor (see below). Out of 12 lineage-specific duplications, six TFs share one or more regulated genes with their paralogs. Combining these results, we hypothesized that little of gene regulation arises by duplication.

#### Ancient paralogs rarely conserve regulation from their common ancestor

In contrast, an analysis by Teichmann and Babu [4] found that '... more than two-thirds of *E. coli *... transcription factors have at least one interaction in common with their duplicates.' More broadly, they report that, '... more than one-third of known regulatory interactions [in *E. coli*] were inherited from the ancestral transcription factor or target gene after duplication.' However, they identified distant homologs within *E. coli *by analyzing structural domains. Most of these structural paralogs diverged so long ago that the homology cannot be identified by protein BLAST (data not shown). Because gene regulation in bacteria evolves rapidly [5,6,17], we suspected that these paralogs diverged before the current regulation of these genes evolved. If this is correct, then these regulatory similarities between paralogs were not inherited from a common ancestor, and might instead be due to convergent evolution.

To determine whether the homologs identified by Teichmann and Babu [4] diverged before their current regulation evolved, we compared the evolutionary ages of the duplication events and of the gene regulation. In particular, we considered whether one of the duplicated genes had been acquired by HGT after the duplication event. If HGT occurred after the duplication event, then because the regulatory relationship cannot predate the coexistence of those genes in the same genome, the regulation must have evolved after the acquisition, and hence after the duplication as well.

**Figure 7:**
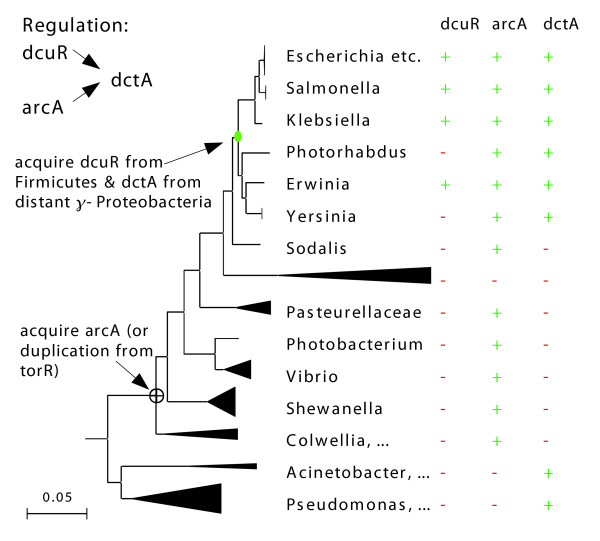
Convergent evolution of regulation of *dctA *by two distantly-related response regulators. From the gene trees (not shown), we identified subfamilies that correspond to *dctA*, *dcuR*, and *arcA*. For example, we split *arcA *and its relatives from the closely related *torR *subfamily of response regulators, which is also present in many γ-Proteobacteria. We show the presence and absence of these subfamilies within the γ-Proteobacteria. The coexistence of *dcuR *and *dctA *in the genome is relatively recent, which shows that this regulation evolved after *dcuR *diverged from *arcA*.

For example, the response regulators *arcA *and *dcuR *(which is also known as *yjdG*) were identified as homologs by Teichmann and Babu [4], and they both regulate *dctA *[27]. As shown in Figure 7, *dcuR *and *dctA *are present in other Enterobacteria but are absent from more distant γ-Proteobacteria such as *Pasteurella*, *Vibrio*, and *Shewanella *spp., which shows that these genes were acquired relatively recently. Because both *arcA *and *dcuR *are more closely related to genes from a variety of distantly related bacteria than they are to each other (data not shown), they must have diverged from each other long before the transfer of *arcA *or *dcuR *into the *E. coli *lineage. Also, although *dctA *is present in some of the more distant γ-Proteobacteria, those lineages lack *arcA*, which shows that these genes were not in the same genome until relatively recently. We conclude that the joint regulation of *dctA *by ArcA and DcuR must have evolved after the transfer of *dcuR *and *dctA *into the *E. coli *lineage, and long after the divergence of *arcA *from *dcuR*.

We repeated this analysis for 30 randomly selected examples of shared regulation between homologous genes from Teichmann and Babu [4] (see Additional data file 4). In most cases we found that one of the genes had been acquired by HGT relatively recently, and from bacteria that do not appear to contain orthologs of the other genes, so that the regulation presumably evolved after the horizontal transfer event. We also identified inconsistent operon structure, which seemed to be evidence against evolution by duplication. For example, the paralogous genes *tdcE *and *pflB *are both regulated by CRP and IHF. Because *tdcE *and *pflB *are in operons, and because the first genes of those operons are not homologous (*tdcA *and *focA*), the regulation of the two operons probably arose independently. Alternatively, the first genes could have inserted between the duplicated genes and their promoters (after the duplication event), but this seems unlikely. Furthermore, changes in operon structure are often accompanied by changes in gene regulation [28]. We confirmed only one of the 30 interactions as evolving by duplication. Thus, most of the regulatory similarities between distant homologs are not inherited from a common ancestor. The pattern that Teichmann and Babu [4] identified might instead reflect convergent evolution.

#### Closer paralogs rarely conserve regulation from their common ancestor

To determine whether closer homologs have a tendency toward shared regulation, we identified homologs within the *E. coli *genome by protein BLAST. We required the score from BLAST to be at least 30% of the self-score for each gene individually. Because this threshold is effective at distinguishing orthologs within the γ-Proteobacteria from other homologs [29], this threshold should select for paralogs within the γ-Proteobacteria. Of the 14,993 homologous pairs of proteins in *E. coli *K12, this rule selected 1,560 pairs. Given these 'close paralogs', and the regulatory interactions between genes and TFs from RegulonDB, we looked for three types of shared regulation between paralogs, as in the report by Teichmann and Babu [4]. We identified paralogous TFs that regulated the same gene (for example, ArcA and DcuR regulate *dctA*; see above), paralogous genes regulated by the same TF (for example, CRP regulates *araE *and *galP*), and paralogous TFs that regulate paralogous genes (for example, CpxR regulates *ompC *and OmpR regulates *ompF*). As above, we excluded autoregulation from consideration. A detailed examination of the interactions is given in Additional data file 5.

Across all three types of shared regulation, we found that 14% of the regulatory interactions in RegulonDB were shared between paralogs (Table 1). After removing regulation that is more recent than the duplication event and removing shared regulation that has inconsistent operon structure, however, it appears that only 5% to 8% of the interactions actually evolved by duplication. (The uncertain 3% represent interactions in which the relative age of the duplication and of the regulation was unclear, and operon structure could not be used to clarify.) The other 6% to 9% of interactions arose by convergent evolution between paralogs.

**Table 1:** Evolution of gene regulation by duplication or by convergent evolution

Type of shared regulation	Interactions (*n*)	Percentage
All three types of shared regulation, combined	425	14.2%
Evolved by duplication	145	4.8%
Unclear	94	3.1%
Convergent evolution	186	6.2%
		
Interactions that are not shared with paralogs	2,570	85.8%
All of RegulonDB (with autoregulation removed)	2,995	100.0%
		
Type 1: paralogous TFs regulate the same genes	212	7.1%
Evolved by duplication	84	2.8%
Unclear	64	2.1%
Relative ages are unclear, and TFs bind the same site	62	2.1%
Duplication of TFs is recent, but TFs bind different sites	2	0.1%
Convergent evolution	64	2.1%
Duplication of TFs is old, and TFs bind different sites	26	0.9%
Duplication of TFs is old, but TFs bind the same site	28	0.9%
Duplication of TFs is old, and sites are not known	10	0.3%
		
Type 2: paralogous genes are regulated by the same TF	290	9.7%
Evolved by duplication	76	2.5%
Unclear	26	0.9%
Convergent evolution	188	6.3%
Differences in operon structure	166	5.5%
Operons are consistent, but acquired after duplication	22	0.7%
		
Type 3: paralogous TFs regulate paralogous genes	54	1.8%
Evolved by duplication (similar ages)	8	0.3%
Convergent evolution	46	1.5%
Complex HGT of regulated genes after TF duplication	16	0.5%
TF duplication precedes that of regulated genes	30	1.0%

For each case of shared regulation between paralogs, we examined the evolutionary histories of the duplicated genes to determine whether the regulation was likely to be conserved from the common ancestor. If so, then the regulatory similarity probably evolved by duplication; if not, then the similarity results from convergent evolution. For cases where two paralogous transcription factors (TFs) regulate the same operon, we also considered whether the TFs bind to the same site. For cases where two paralogous genes are regulated by the same TF, we also considered whether the first genes in the operons were homologous, as would be expected for evolution by duplication. The results are tabulated here (see Additional data file 5 for individual interactions). Because some regulatory interactions are shared with paralogs in more than one way, the totals are smaller than the sums over the types.

One mechanism of convergent evolution was apparent; we found four operons that were clearly acquired after the duplication of their regulators, and yet each of these operons are regulated by paralogous TFs that bind to shared sites (the paralogous TFs that share binding sites are *gntR*/*idnR *and *narL*/*narP*). Apparently, if paralogous TFs maintain overlapping DNA binding specificities, then a single site can evolve to bind both TFs. As the evolution of these sites relies on the functional similarity of the paralogs, it is debatable whether these cases should be termed convergent evolution. In most cases, however, no such simplifying mechanism was apparent, and we believe that the paralogs evolved similar regulation entirely independently.

To determine whether the amount of shared regulation between close paralogs was greater than would be expected by chance, we randomly shuffled the regulatory network 1,000 times (see Materials and methods, below). All 1,000 shuffled networks had fewer cases of regulatory similarity between paralogs than were found in the true network. When we considered each type of sharing separately, we found the same result. In particular, paralogous TFs regulate paralogous genes significantly more often than we would expect by chance, whereas a previous report found it to be less common than expected [4]. To determine whether convergent evolution was more common than expected by chance, we compared the regulatory similarities in the shuffled networks with the number of regulatory similarities between paralogs that evolved independently. We found that all three types of convergent evolution occurred more often in the real network than in any of the shuffled networks. Thus, convergent evolution appears to be a significant factor in the evolution of gene regulation.

We also considered autoregulation separately. A recent report [30] found a weak but statistically significant similarity in autoregulation within families of TFs. However, among the close paralogs, we did not find any similarity between paralogs in their tendency to autoregulate. More precisely, we considered pairs of close paralogs of TFs and we considered whether autoregulation was correlated for these pairs. We did not find an effect (odds ratio 1.15; *P *> 0.5, by Fisher's exact test; 66 pairs). Again, the pattern that was identified in the previous work that considered more distant paralogs could possibly result from convergent evolution.

Overall, we found that only 5% to 8% of regulatory interactions arose by duplication within the *E. coli *lineage. Another 6% to 9% of regulatory interactions reflect independent (convergent) evolution of similar regulation for homologous genes. Thus, convergent evolution probably accounts for more of the regulatory interactions than does evolution by duplication. One caveat in our analysis is that these proportions can be expected to rise as more knowledge of the *E. coli *regulatory network becomes available. Missing information from either of two paralogs will cause any duplication of regulation to be missed, and so the amount of duplicate regulation that can be identified grows more rapidly than the size of the network. However, because only 13% of the TFs evolved by duplication within the *E. coli *lineage, and because the majority of the regulatory similarities between paralogs reflect convergent evolution, we can still conclude that little of gene regulation has evolved by duplication.

#### Complex regulation of acquired genes

Although most TFs were acquired by HGT, we also found that most of the global regulators are more ancient. Because the 20 global regulators account for about two-thirds of the regulatory interactions in RegulonDB, we wondered how these global regulators relate to the bulk of *E. coli *genes, which have been acquired by HGT.

**Figure 8:**
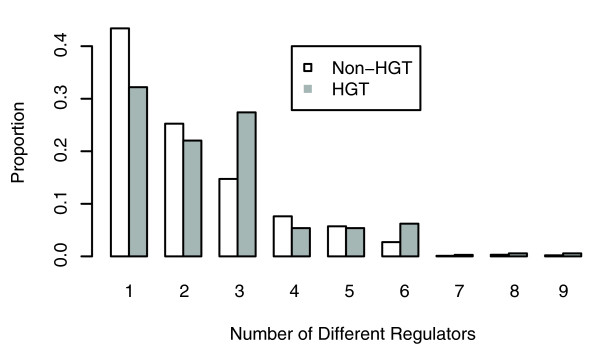
Complex regulation of horizontally acquired genes. Horizontal gene transfer (HGT) genes were identified by an automated presence/absence method, and the number of different regulators for each gene was taken from RegulonDB. Genes without any known regulation were not included. HGT genes tend to have more regulators than other genes (*P *< 10^-4^, by Wilcoxon rank sum test; 354 HGT genes and 998 other genes).

Because many of the genes in *E. coli *were acquired by HGT relatively recently, we hypothesized that these genes would have less time to evolve complex regulation. In particular, we expected that HGT genes would tend to be regulated by fewer TFs than other genes. However, when we examined the regulation (as described in RegulonDB) of HGT genes that were identified by the automated presence/absence approach, we found that HGT genes are significantly more likely than other genes to be regulated by several different TFs (Figure 8). For example, 68% of HGT genes are regulated by two or more TFs, but only 57% of the other genes in RegulonDB are regulated by multiple TFs (*P *< 0.0005, by Fisher's exact test). We also compared HGT genes with conserved γ-Proteobacterial genes that are reported not to undergo HGT [29], and we again found that the HGT genes had, on average, more complex regulation (data not shown).

When we examined the HGT genes that are regulated by two or more TFs, we found that 30% of them are regulated by both an adjacent neighbor regulator and by a global regulator. The global regulator is usually CRP (61/73 cases). HGT genes are preferentially regulated by CRP; of the genes with characterized regulation in RegulonDB, CRP regulates 48% of the HGT genes but only 23% of the other genes (*P *< 10^-15^, by Fisher's exact test). This presumably occurs because CRP regulates carbon source choice and because many of the HGT genes encode the catabolism of specific carbon sources. (At a false discovery rate of 5%, none of the other global regulators has a statistically significant association with HGT genes.) More generally, we speculate that HGT genes are particularly likely to be niche specific and hence to require complex regulation. In any case, these results suggest that the evolution of regulation is driven by selection and that it evolves more rapidly than the time scales considered here.

#### Regulation of acquired genes: evolving new sites versus acquiring genes with regulatory signals

Given that most of the global regulators are highly conserved within γ-Proteobacteria, and that typical genes are preferentially transferred within the γ-Proteobacteria, we wondered whether these genes would conserve their regulation across HGT events. Among the genes that have been acquired by the *E. coli *lineage from other γ-Proteobacteria, we examined neighbor regulated operons, CRP-regulated genes, and genes that are regulated by global regulators and have identifiable binding sites.

To determine whether genes are acquired together with regulatory signals, we first considered neighbor regulated operons that have undergone co-transfer with their regulators within the γ-Proteobacteria. In these cases (17 of the 39 neighbor regulators that we examined), it is likely that the regulation of the operon by the adjacent TF predates the horizontal transfer event. For six of these 17 operons, there is another known regulator for the operons, and in five of those cases that regulator is CRP. CRP is conserved in both sequence and DNA-binding specificity across the γ-Proteobacteria; for example, the protein Clp from the distant γ-Proteobacterium *Xanthomonas campestris *is 45% identical to *E. coli *CRP, has a similar DNA-binding specificity, and complements a *crp *knockout when cloned into *E. coli *[31,32]. So, we used a position-specific weight matrix derived from known CRP binding sites in *E. coli *to predict binding sites for CRP upstream of these operons and upstream of their xenologs in other γ-Proteobacteria (see Materials and methods, below). We found likely binding sites upstream of xenologs for three of the five operons (Table 2). We did not find CRP sites upstream of *E. coli melAB *or its xenologs, perhaps because CRP does not bind this promoter in the absence of *melR *[33]. Finally, *dsdXA *has a conserved CRP binding site in Enterobacteria, but the xenolog from *Photobacterium profundus *does not. Overall, this analysis suggested that complex regulation, which in these cases involved both a neighbor regulator and CRP, can be maintained across HGT events.

**Table 2:** Binding sites for CRP upstream of *Escherichia coli *operons and their xenologs

Operon	Organism	Position	Score	Site sequence
yiaKLMNOPQRS	*E. coli *K12	-175	9.1	aAgTGTGccgtagtTCACgaTc
yiaKLMNOPQRS	*Haemophilus influenzae *RD KW20	-148	10.3	aAaTagGAtctagaTCACAaaa
araBAD	*E. coli *K12	-131	9.1	ttaTtTGcacggcgTCACAcTt
araBDA?C	*Vibrio parahaemolyticus *RIMD 2210633	-177	6.3	tggaGTtcgatgagagcCggTt
		-137	6.5	cgacaTGAtgacgacgAtcgcc
gntKU	*E. coli *K12	-171	13.1	aAaTtTGAagtagcTCACAcTt
gntK-edd	*V. cholerae*	-131	11.5	gttTGTGttatagcTCACAtTt

These *E. coli *operons are regulated by CRP as well as by an adjacent regulator and have been co-transferred, together with their neighbor regulators, between the *E. coli *lineage and other γ-Proteobacteria. We used a weight matrix to identify potential binding sites for CRP upstream of these operons and their xenologs. For each site we report its sequence, its score in bits, and its position relative to the start codon of the first gene in the operon. The sites that were used to build the weight matrix have 8.41 ± 2.66 bits (mean ± standard deviation). Within each site's sequence, positions that match the consensus nAnTGTGAnnnnnnTCACAnTn are capitalized.

We then examined the CRP regulon more broadly. As discussed above, CRP regulates a larger proportion of HGT genes than of native genes. Although *crp *has evolutionary orthologs only within β,γ-Proteobacteria (data not shown), most of the HGT genes that are regulated by CRP (81%) have their best hits to more distantly related bacteria. We examined a random sample of 20 of these genes that were putatively acquired from distant bacteria by hand, and we confirmed that most of them (18/20) were acquired from distantly related bacteria. Many of these genes (12/20) have a sporadic distribution of homologs in intermediate related bacteria such as *Vibrio *spp., which suggests that there might be a more recent HGT event as well. In this case, we wondered whether the regulation occurred before or after this intermediate HGT event. When we searched for CRP sites upstream of the first gene in the operon in these intermediate species, we found likely regulatory sites for four out of 12 genes. Thus, in most cases, these genes have evolved regulatory sites for CRP after their transfer into the *E. coli *lineage, even if they were acquired from other γ-Proteobacteria. Given that the CRP regulon is the largest in *E. coli*, it is striking that most of this regulation has evolved relatively recently.

We also considered whether other global regulators have binding sites that have been conserved across HGT events within the γ-Proteobacteria. We considered *E. coli *genes that were acquired from other γ-Proteobacteria (according to our automated presence/absence analysis), that are regulated by global regulators, that are the first gene of their operon, and that have upstream matches to weight matrices from DPInteract [34]. We found 20 genes that matched these criteria, and in just six cases the closest xenolog also has a potential site for the regulator. Because we used a weak threshold to identify sites (6.0 bits), this could be an overestimate. This analysis confirmed that many of the binding sites for global regulators have evolved relatively recently.

Finally, according to our automated analysis, 57% of the HGT genes in *E. coli *were acquired from outside the β,γ-Proteobacteria. Because most *E. coli *TFs do not have orthologs in such distantly related bacteria [5,17], most of this regulation probably evolved after the transfer event. Overall, we found a few cases in which complex regulation has been conserved across HGT events, but most of the regulation of these HGT genes in *E. coli *appears to have evolved after the genes were acquired.

## Conclusion

We have shown that the TFs of *E. coli *evolved primarily by HGT rather than by duplications within the *E. coli *lineage. Lineage-specific duplication accounts for a small minority of TFs (13%) and for an even smaller proportion of regulatory relationships (5% to 8%). In contrast, most of the TFs (64%) have been acquired by HGT after the divergence of the *E. coli *lineage from *Shewanella *spp. These findings support the model of 'allopatric gene divergence', wherein a TF's function diverges after HGT moves the TF into a new genome with new selective pressures, and once the TF's function diverges it is reacquired [9]. For example, *dcuR *and *arcA *(Figure 8) appear to be allopatric paralogs. Allopatric divergence avoids the complications of selection for both copies of the gene that arise when two new paralogs are in the same genome. One might imagine that, once reunited in the same genome, there would be crosstalk or conflict between these regulators, but this is not generally the case. Indeed, even for TFs that underwent duplication within the *E. coli *lineage, only about half of them share binding sites with their paralogs. DNA binding specificity may evolve rapidly; many TFs are neighbor regulators that bind just one or two sites in the genome, so that their DNA-binding specificity should not be highly constrained by selection. Paralogous TFs usually respond to different signals as well, but we do not address that here.

We found that TFs are often co-transferred with their regulated genes, which confirms a suggestion [14] that neighbor regulation is maintained by HGT. Thus, neighbor regulators can be viewed as being 'selfish regulons', as an analog to the selfish operon theory [35,36]. More precisely, we imagine that the genes themselves and the regulatory relationship between them benefit the host, but the proximity itself may not be of benefit to the host and is selected for by HGT. It remains unclear how neighbor regulation arises in the first place; we discuss that issue below. We found that many of the putative, as yet uncharacterized TFs of *E. coli *have also been co-transferred with adjacent genes, and so we infer that most of these TFs are also neighbor regulators and that they also regulate just one or two operons [14].

Although most TFs have been acquired by HGT, most of the global regulators are well conserved within the γ-Proteobacteria. Because these global regulators are responsible for about two-thirds of known regulation, gene regulation could be more conserved than would be implied by the recent origins of the typical TF. However, HGT genes have more complex regulation than do native genes, and most of these HGT genes are acquired from distant bacteria in which global regulators are not conserved. Even for genes that were acquired from other γ-Proteobacteria, most of the binding sites for global regulators that are found in *E. coli *appear not to be conserved across the HGT events. Thus, it appears that on the time scales considered here, regulation evolves rapidly, even though the global regulators evolve slowly.

### Nonrandom evolution of gene regulation

We found two nonrandom patterns in the evolution of gene regulation. Both of these patterns appear inconsistent with neutral or nearly neutral theories for the evolution of gene regulation. First, although regulatory similarities between paralogs (either paralogous TFs or paralogous regulated genes) account for 14% of the regulatory interactions, evolutionary analysis shows that these similarities often result from convergent evolution rather than being conserved from the common ancestor. The tendency toward convergent evolution is statistically significant. We propose that paralogs tend to have similar (but distinct) functions, and that selection sometimes causes these paralogs to have similar regulatory interactions. For example, the distant paralogs *aroF *and *aroG *encode isozymes with different feedback inhibition, but both genes are regulated by TyrR. The distant paralogs *phoE *and *ompC *encode outer membrane porins with different specificities, and both are regulated by two-component systems that sense ion concentrations (PhoB/PhoR and EnvZ/OmpR). We also found a few cases in which a new site has evolved to bind two paralogous TFs that have overlapping DNA binding specificities.

Second, HGT genes tend to be under more complex regulation than native genes, which is surprising. HGT genes have had less time to evolve complex regulation. Also, HGT genes tend to be less highly expressed than native genes (*P *< 10^-15^, by Wilcoxon rank sum test; expression levels from Price and coworkers [37]), which implies weaker selection on their regulation. We propose that many HGT genes are niche specific and hence require more complex control, whereas native genes are (relatively) constitutively expressed. In particular, many of the neighbor regulated genes are also regulated by the catabolite repressor CRP, so that each gene's expression is regulated by the availability of glucose as well as by a more specific signal related to the gene's function. More generally, HGT genes may be 'second best' systems that are not needed under optimal conditions, and hence need to respond to global regulators as well as to a specific sensor. In contrast, native genes may be regulated by a single sensor. Because our knowledge of gene regulation in *E. coli *is highly incomplete, however, we cannot rule out the possibility that the apparently complex regulation of HGT genes results from some bias in what geneticists choose to study.

### Neighbor regulators as 'selfish', niche-specific regulons

The mechanism by which neighbor regulators form remains unclear. If we examine the closest homologs of neighbor regulators and regulated genes that are not near to each other, then we usually find that these homologs are not in the same genomes (data not shown), and so the proximity does not appear to result from deleting intervening genes. We also note that neighbor regulated genes are more likely than other characterized genes to be in operons instead of transcribed individually (*P *< 0.01, by Fisher's exact test), so there may be some operons that are evolving 'selfishly' along with their regulators, even though the selfish model does not appear to apply to operon formation in general [25,28,38,39].

We speculate that neighbor regulation might arise because it allows the TF to bind to a single site and regulate both the TF and the regulated operon. For example, the majority of TFs regulate their own transcription, and if an HGT event inserts an operon adjacent to the TF, then the pre-existing site could regulate that operon's transcription. This would explain why the majority of neighbor regulators are divergent from their regulated genes, and strong selection to maintain the shared site might explain why the divergent orientation is associated with autoregulatory TFs [14,15]. The other neighbor regulators might arise from divergent neighbor regulators by local inversion, as can be seen for *xapR *(Figure 3).

Another explanation is that neighbor regulation might be selected for because a newly synthesized TF would be closer to its target [14]. This type of proximity could also explain why neighbor regulators tend not to be transcribed in the convergent orientation relative to the regulated operon, because the convergent orientation increases the distance from the newly synthesized TF to its site by a few kilobases [40]. However, the time for TFs to find their targets is short regardless of their location; TFs bind to specific sites at rates of around 10^8^/M per second, and if the TF has a single site in the genome then that site's concentration is about 10^-9 ^M, so that a newly synthesized TF should find its binding site, anywhere in the genome, in around 10 seconds on average [41]. The search time might be greater because of nonspecific binding to DNA [40], but *in vivo *the lactose repressor finds its target in at most a few minutes [42]. Thus, we doubt that there is selection for a TF to be encoded near to its target site(s).

Regardless of the origin of neighbor regulation, the repeated HGT of neighbor regulators within γ-Proteobacteria suggests that these regulons are niche specific. Niche-dependent selection for these genes is also consistent with the functional bias of HGT genes [23], the role played by HGT genes in peripheral (nonessential) rather than central metabolism, and the metabolic compatibility of acquired genes with the pre-existing capabilities of the host [43]. Conversely, the sporadic distribution of these genes is consistent with the high rate of loss of recently acquired genes [44]. The rapid loss would most likely be neutral, but it could also reflect selection against capabilities that are deleterious if not frequently needed [45].

### Complex patterns of HGT

We found that HGT of TFs is rampant, and that many genome sequences are required to detect these events, so that the absence of the gene from intermediate groups of bacteria is clear. Because of HGT between related bacteria, simply comparing the gene tree with the species tree (for those species that contain the gene) may not be a sensitive indicator of HGT. We found that HGT of global regulators was rare, but because these regulators are resistant to gene loss we cannot use gene absence to help us identify HGT. Thus, we could be underestimating the rate of HGT for these genes. As in the case of *crp*, these global regulators often have conserved context, so insertion of a xenolog and loss of the original gene appears not to occur. However, homologous recombination could be replacing all or parts of these sequences in place, especially given the high conservation of these genes (for example, the DNA sequence of *crp *is 88% identical between *E. coli *and *Salmonella typhimurium *LT2). Indeed, some workers argue that all bacterial genes are subject to frequent HGT events [46]. In this case, the distinction between HGT and other genes might not be meaningful, but there remains a difference between genes that are frequently gained and lost (niche-specific neighbor regulators) and genes that have occasionally undergone recombination (global regulators).

Why should HGT between related bacteria be prevalent? One possibility is that DNA from related organisms is more easily integrated into the host's genome. In general, however, the divergence of the genes involved appears to be too great for homologous recombination. Another possibility is that related bacteria are more likely to have genes that fit into the pre-existing metabolic pathways of the new host, which increases the likelihood of HGT [43]. Finally, our results suggest that compatibility of gene regulatory systems might select for HGT between related bacteria. Even when genes are acquired together with neighbor regulators, these genes are also often regulated by global regulators such as CRP, and we found some cases in which CRP binding sites were conserved across transfer events. CRP and most of the other global regulators from *E. coli *are not present in distant bacteria [17], and so the transfer of regulatory sites can only occur between related bacteria. Even if the operon has only one regulator, differences in the core transcriptional machinery in different hosts might prevent the newly acquired neighbor regulator from functioning, especially for activators.

## Materials and methods

### Regulatory interactions

We obtained regulatory interactions from RegulonDB 5.6 [1]. After removing RNA genes and pseudogenes, and the housekeeping sigma factor RpoD, we had 159 characterized TFs, 1,354 regulated genes, and 3,085 regulatory interactions between them. A few of the TFs are heterodimers; in these cases, we analyzed only one of the two subunits. We also examined TF and gene annotations in EcoCyc [47] and known operons in RegulonDB.

### Evolutionary histories of TFs

We investigated the evolutionary histories of TFs by comparing the gene tree with the species tree. As a first step, we used fast neighbor-joining trees [48] for COGs, PFams, and *ad hoc *BLAST families from the MicrobesOnline tree browser [49] and we compared the gene trees to the MicrobesOnline species tree. (The most relevant parts of the species tree are shown in Figure 2, and the construction of the species tree is described below.)

Given a gene tree and a species tree, we identified horizontal transfer events using a combination of the gene phylogeny and the pattern of gene presence and gene absence. If a strongly supported clade in the gene tree was present in disparate genomes, so that three or more deletion events would be required to explain the distribution of the subfamily on the species tree, then we assigned an HGT event. Deletions in the highly reduced genomes of the insect endosymbiont group (*Buchnera*, *Wigglesworthia*, and *Blochmannia*) were not considered as evidence for HGT. Given that HGT appears to be common in bacteria, the threshold of three or more deletion events is conservative. In particular, with higher thresholds, a large number of deletions from ancestral bacteria are required to explain the present distribution of genes, which requires the ancestral bacteria to have had unreasonably large genomes [50,51].

If the gene tree showed paralogs, and the phylogeny of two subgroups was consistent with the species tree, then we assigned a gene duplication event. Histories that did not meet either of these criteria were considered native, even if there were minor discrepancies between the species tree and the gene tree. If a gene exhibited evidence for both HGT and duplication, then we used the most recent event to classify the gene's origin (for example, *purR*/*rbsR*, in Figure 4, is classified as a duplication).

Once we had a tentative classification, we confirmed it by checking for close homologs (by BLASTp) that might be absent from the gene family (because of the limitations of gene family assignment) and by building a smaller and more accurate phylogenetic tree for a selected subset of homologs. To build these higher quality trees, we used MUSCLE [52] to align the protein coding sequences, Gblocks to trim the alignments [53], and both TreePuzzle [54] and phyml [55] to build phylogenetic trees.

We also asked whether the putative HGT event affected the *E. coli *lineage. For example, as seen for *crp *(Figure 5), the tree suggests a transfer event from *E. coli*'s ancestors to another lineage, but this does not imply that *E. coli*'s ancestors acquired the gene by HGT. These genes were classified as native.

We assume that these genes were transferred from other bacteria into the *E. coli *lineage, rather than *vice versa*, even though it is theoretically possible that these TFs arose in the *E. coli *lineage relatively recently and were then transferred elsewhere. Because most of the TFs belong to large families that are present in many other bacterial lineages, and also because these TFs often have distant paralogs in *E. coli*, a recent origin of these families within the *E. coli *lineage is not plausible.

### Species tree

The species tree was computed from maximum likelihood trees of concatenated proteins by using matrix representation of parsimony [56]. The maximum likelihood trees were generated from a lower quality guide tree by selecting, for each internal node in the guide tree, a small number of descendant genomes and close out-groups (less than 20 genomes in total). Given this small group of genomes, we identified COGs [22] that are present as a single copy in each genome. Because these groups of genomes usually consisted of close relatives, there were typically hundreds of conserved genes. We aligned and trimmed each COG, again using MUSCLE and Gblocks, and concatenated the alignments. Because the resulting alignments were often very large, we removed invariant sites, and if the alignment still contained over 5,000 positions then we took a random sample of sites. We then built a tree with phyml, using four categories of evolutionary rates. We converted the trees to a matrix of characters [56] and used PAUP 4.0b10 [57] to infer the most parsimonious tree. Finally, we used PHYLIP [58] to infer maximum likelihood branch lengths, with gamma-distributed rates, from a concatenated alignment of 74 highly conserved proteins.

A fuller description of the species tree construction is available online [49]. The tree does not contain bootstrap values, but most of the source trees have strong bootstrap support and are congruent with each other (data not shown). The most relevant uncertainties are the placement of *Photorhabdus*, and whether *Sodalis *should be grouped with the other insect endosymbionts (*Buchnera *and so on).

### Sampling of regulators

We examined all of the top 20 global regulators, which account for about two-thirds of the regulatory interactions in RegulonDB. For neighbor regulators, we examined those that were described in an earlier compilation of regulatory interactions, ColiNet 1.1 [59], which we used in the initial phase of this project. Although this is not a truly random sample, we do not know of any reason why the more recently characterized regulators would have different evolutionary histories. We examined a random sample of 23 of the other characterized regulators in RegulonDB. Again, these were primarily regulators that were described in ColiNet.

We identified putative regulators in *E. coli *K12 by searching for gene ontology GO:0003700 ('transcription factor activity') using the MicrobesOnline database. We randomly selected 20 of these to examine, and we verified that they were predicted to contain helix-turn-helix domains (by using InterPro), that they were not annotated as restriction enzymes or DNA modification enzymes, and that they were not already characterized according to EcoCyc [47].

### Automatic identification of HGT genes

**Figure 9:**
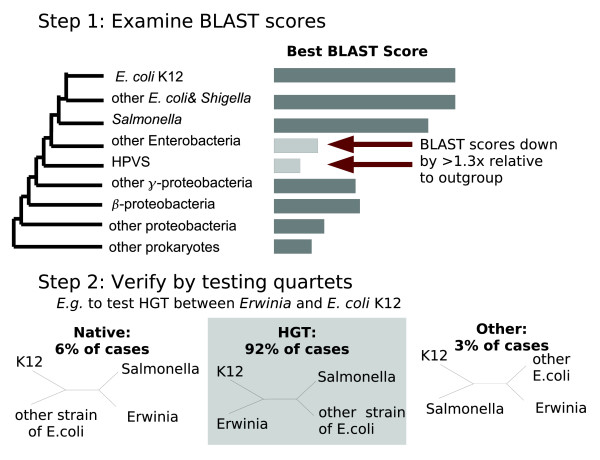
Automated identification of HGT genes. We examined the highest Basic Local Alignment Search Tool (BLAST) scores of homologs within groups of genomes at increasing distances from *Escherichia coli*. If the BLAST score was substantially lower (by a factor of 1.3) in two consecutive groups relative to its best score in more distant genomes, then the gene was considered a candidate for horizontal gene transfer (HGT). Given such candidates, we then used a quartet test to determine whether the best hit from the more distant genome was actually more closely related to the *E. coli *gene than were the best hits from intermediate genomes. The quartet test confirmed HGT in 92% of these cases, and for 71% of the genes whose quartet topology indicated HGT the topology was strongly supported (*P *< 0.05, by Shimodaira-Hasegawa test in tree puzzle [54]). 'HPVS' refers to *Haemophilus*, *Pasteurella*, *Vibrio*, *Shewanella*, and related species.

To identify HGT automatically, we looked for genes that lack close homologs in consecutive groups of related bacteria (Figure 9). We defined 'close' homologs by BLAST scores, and to confirm the putative HGT we used a quartet test (see Figure 9). This approach contrasts to approaches that rely heavily on the gene tree [13,24] and is more similar to presence/absence analyses [60,61]. Although the method is conservative, and misses many HGT events (data not shown), it classifies about a quarter of protein coding genes in *E. coli *K12 as HGT, which yields a sufficiently large sample for analysis.

The quartet test was not conducted if there was no more distant homolog in each of the groups of genomes that were 'missing' good hits to the gene, because in these cases we do not have four genes to form a quartet from. If we did have a gene from each group of genomes, we aligned the four genes with MUSCLE, we removed positions with gaps and we tested the likelihood of all three topologies with tree puzzle [54], using gamma-distributed evolutionary rates.

### Shuffled regulatory network

To test whether the regulatory similarities between paralogs occurred more often than we would expect by chance, we used a simple null hypothesis that the regulatory network evolves randomly. This null hypothesis is equivalent to a simplistic neutral model in which binding sites for regulators arise neutrally, and binding sites for global regulators arise more frequently than for other regulators, so that they regulate more genes.

To test this null hypothesis, we shuffled the network so that that the number of interactions for each TF and for each regulated gene was unchanged (similar to the report by Maslov and Sneppen [62] but for regulatory networks). More precisely, we selected the regulated genes for each TF by sampling without replacement from the complete set of regulated genes. We re-sampled parts of the network to avoid duplicate interactions between regulated genes and TFs. This gave networks with the same degree distribution as the original network, both for TFs and for regulated genes.

An alternate randomization test is to permute the paralogy relationships instead of the regulatory networks. (See the report by Teichmann and Babu [4], although they use the terminology of 'domain architectures' rather than paralogy.) This test confirmed that convergent evolution is more common in the real network than expected by chance; all three types of convergent similarity in Table 1 were more common in the real network than in 999 or more of the 1,000 paralogy shuffles that we ran.

### Predicting binding sites for global regulators

We obtained characterized CRP binding sites in *E. coli *from DPInteract [34]. We aligned these sites with MEME [63], converted the alignment to a weight matrix with palindromic symmetry, and used patser [64] to search for sites. We searched from -200 to +100 relative to each gene's start codon, and we considered only potential sites with a score of 6.0 bits or higher. This cut-off is quite weak and leads to high sensitivity but modest specificity; we found sites in *E. coli *for 13 of the 16 CRP-regulated genes that we examined, but 13% of randomly selected upstream regions for xenologs of *E. coli *genes had a hit at 6.0 bits or above. Nevertheless, the xenologous CRP sites in Table 2 are unlikely to have occurred by chance; *yiaK *and *gntK *have hits over 10 bits, which occurs in less than 1% of upstream regions, and *araB *has two nearby sites, which suggests cooperative binding and is also unlikely to occur by chance.

Analyses for other global regulators that have weight matrices in DPInteract were conducted similarly, but without forcing the weight matrix to be palindromic. Some of the sigma factors have multiple models, in which case we used the best score for any model. The weight matrices for lrp and fis were not used because they have poor specificity [34].

## Abbreviations

BLAST, Basic Local Alignment Search Tool; COG, cluster of orthologous groups of proteins; HGT, horizontal gene transfer; TF, transcription factor.

## Authors' contributions

MNP conceived the project and collected the data. PSD provided analytical tools. All authors analyzed the data and wrote the manuscript.

## Additional data files

The following additional data are available with the online version of this paper. Additional data file 1 provides the classification of each TF that we examined, both by evolutionary history and by function. Additional data file 2 plots the sequence conservation of regulators against the number of genes that they regulate. Additional data file 3 illustrates the preference for HGT between related genomes. Additional data file 4 provides an evolutionary analysis of 30 cases of shared regulation between homologous genes from the report by Teichmann and Babu [4]. Additional data file 5 provides an evolutionary analysis of each case of shared regulation between 'close' paralogs.

## Supplementary Material

Additional file 1 Provided is the classification of each TF that we examined, both by evolutionary history and by function. Click here for file

Additional data file 2 The figure plots the sequence conservation of regulators against the number of genes that they regulate. Click here for file

Additional data file 3 The figure illustrates the preference for HGT between related genomes. Click here for file

Additional data file 4 Provided is an evolutionary analysis of 30 cases of shared regulation between homologous genes from the report by Teichmann and Babu [4]. Click here for file

Additional data file 5 Provides an evolutionary analysis of each case of shared regulation between 'close' paralogs. Click here for file
