# Supplementary material for: Horizontal gene transfer and the evolution of transcriptional regulation in Escherichia coli
Source: Genome Biol. 2008 Jan 7;9(1):R4. doi: 10.1186/gb-2008-9-1-r4 (PMC2395238; doi:10.1186/gb-2008-9-1-r4)
Supplement: Additional file 1 — Provided is the classification of each TF that we examined, both by evolutionary history and by function. [file gb-2008-9-1-r4-S1.pdf]

Note S1: Histories of individual TFs

Unless otherwise indicated, all TF functions are taken from RegulonDB (Salgado et al., 2006).

Global regulators

We examined the top 20 global regulators, as defined by the number of different genes that they are reported to regulate in RegulonDB.

Native past *Shewanella* (ancient origin in γ-Proteobacteria): arcA, crp, fis, fnr, fur, ihf (himA/himD), lrp, phoB, rpoE, rpoH, rpoN, rpoS

Native at least to *Shewanella* (previous history is unclear): cpxR, modE, narP, ntrC

HGT: flhDC, fliA, narL

Duplicated with shared regulon members ("from" gives the name of a paralog; the direction is not meaningful): hns (from stpA)

Other duplicates: none

Potential complications in the histories of "native" regulators:

- Fur is absent from the insect endosymbiont group (*Blochmannia*, *Buchnera*, *Wigglesworthia*). *Haemophilus* fur may be deeply branching (HGT), but the tree is poorly resolved because fur is so conserved. Also, *Shewanella frigidimarina* has a second (diverged copy).
- Lrp may have been transferred between *Pseudomonas* and *Bordetella*. Also, lrp appears not to be a global regulator in *Haemophilus influenzae* (D. Friedberg et al, J. Bacteriol. 183:4004-11), so even though lrp is evolutionarily ancient its role as a global regulator may have evolved more recently.
- ModE appears to be native to *Shewanella*, although there is a moderately supported grouping of *Shewanella* with Photobacterium that is incongruent the species tree. (ModE is not present in *Vibrio*.)
- NtrC (also known as glnG) seems to have been transferred from *Shewanella* to *Colwellia*, but as this does not affect the *E. coli* lineage, the gene was classified as native.
- RpoS is not present in *Sodalis*, the insect endosymbiont group, or the *Haemophilus*/*Pasteurella* group. Because these are all reduced genomes, we did not consider this to be strong evidence of an HGT event.

More detail on the instances of HGT & duplication:

- FlhDC is not a neighbor regulator, but has been co-transferred with the adjacent chemotaxis genes motAB. These genes are functionally related, but are apparently regulated by fliA rather than by flhDC. FlhDC also seems to have been transferred as part of the larger flagellar system, including genes that are regulated by flhDC such as fliA and fliC, even though those genes are not nearby in *E. coli.*

- FliA is within a large cluster of flagellar genes and chemotaxis genes. Trees for fliA and for other genes in the cluster consistently group Enterobacteria with *Azotobacter* (a distantly-related γ-Proteobacterium) and with β-Proteobacteria.

- Hns is reported to regulate some of the same genes as stpA, but because of the low expression of stpA in wild-type cells, this might not be physiologically relevant, and stpA may act primarily as an RNA chaperone (Zhang et al., 1996, EMBO J. 15:1340-9).

- NarL duplicated from narP within γ-Proteobacteria (but before the divergence of *E. coli* from *Shewanella*), and also appears to be HGT between some *Shewanella* species and a sub-group of Enterobacteria, because the narL version of the gene is absent from Vibrionaceae, from Pasteurellae, from the insect endosymbiont group that includes *Buchnera*, from *Sodalis glossinidius*, and from other Shewanellas. Because of this transfer and because narL is also heterogeneously present in more distantly-related γ-Proteobacteria, narL is classified as repeated HGT within γ-Proteobacteria. Also, narL has been co-transferred with its sensor kinase narX, and probably with its regulated gene narK as well. Despite these HGT events, narP and narL are clearly ancient paralogs, and their regulons overlap considerably.

Some of these global regulators are also neighbor regulators (In Figure 6, these were included under the category of global regulators, and not under the category of neighbor regulators.)

- CpxR is a response regulator that is co-transcribed with its histidine kinase cpxA. The cxpRA operon is divergent from cpxP, which is regulated by cpxR and which inhibits the activity of cpxA (and hence, indirectly inhibits cpxR, forming an autoregulatory feedback loop).

- CRP represses its own transcription and activates that of a divergent transcript that encodes a short RNA; this seems to be an antisense mechanism for CRP auto-regulation (K. Okamoto et al., J. Bacteriol. 170:5076).

- FliA regulates many flagellar genes that are colocated with fliA, including the adjacent transcript fliC.

- ModE regulates the nearby operon modABC, but because of the intervening gene b0762, which appears to be a recent insertion, modE is not, strictly speaking, a neighbor regulator.

- NarXL is adjacent to the regulated gene narK.
- NarP is adjacent to its regulated operon napFDAGHBC-ccmADBCDEFGH.

Neighbor Regulators

After excluding 5 neighbor regulators that were also global regulators (cpxR, crp, fliA, narL, and narP), we examined 39 of the remaining 67 neighbor regulators, primarily those that were also present in an earlier database, ColiNet.

Native: dcuR, glpR, ilvY, leuO, nhaR

Co-transfer and repeated HGT within γ-Proteobacteria ("with" gives the regulated genes that were transferred along with the TF): araC (with araB), asnC (with asnA), betI (with betT), cadC (with cadBA), cynR (with cynTSX), dsdC (with dsdXA), gntR (with gntKU), hcaR (with hcaEFCB), idnR (with idnK), lacI (with lacZ), lysR (with lysA), melR (with melAB), rtcR (with rtcAB), xapR (with xapAB), yiaJ (with yiaKLMNO-lyxK-sgbHUE)

Co-transfer only: atoC (with atoDAE), csgD (with csgBA), ebgR (with ebgAC), glcC (with glcDE-FGB), malI (with malXY), mhpR (with mhpCDF), pspF (with pspA), torR (with torCAD), treR (with treBC)

Repeated HGT only: malT, soxR

Other HGT: rpiR

ORFan: caiF, tdcR

Duplicated with shared regulon members ("from" gives the name of a paralog; the direction is not meaningful): exuR (from uxuR), galS (from galR)

Other duplicates : acrR (from envR), adiY (from envY and others), evgA (from bglJ)

Complications:

- GntR and idnR duplicated within the γ-Proteobacteria, but both genes also show a complex pattern of presence and absence, which suggests HGT of gntR along with gntKU, or idnR along with idnK, between various γ-Proteobacteria after the duplication event. Hence both genes are classified as co-transferred/repeated-HGT above, rather than as duplicated. Nevertheless, the genes do reflect an older duplication, and the regulons overlap.
- The tree for ilvY suggests HGT between Enterobacteria and Shewanella, but ilvY is present in almost all of the intermediate genomes and the tree for the adjacent regulated gene ilvC

shows vertical descent, so we classified ilvY as native. It is possible that ilvY was modified by homologous recombination.

- MalT has been transferred with malS, but those genes are shuffled apart in *E. coli*, and so it is not classified as co-transferred.

We also classified the co-transferred neighbor regulators by their mode of action:

- Activators: atoC, cadC, csgD, glcC, mhpR, pspF, xapR
- Repressors: betI, ebgR, gntR, lacI, malI, rtcR, treR, yiaJ
- Both: araC, asnC, cynR, dsdC, hcaR, idnR, lysR, melR, torR

We had roughly equal numbers of each class amongst the co-transferred neighbor regulators (7, 8, 9). Similarly, when considering all regulators, we had roughly equal numbers (50, 51, 58). Thus, there is no relationship between mode of action and co-transfer.

Other Characterized Regulators

We examined a random sample of 23 out of 69 characterized non-global non-neighbor regulators.

Native: birA, fabR, dnaA, nsrR, oxyR, pdhR

HGT: alpA, cueR, cytR, deoR, gutM, hipB, hupA, kdgR, nac, rcsB

ORFan: hycA

Duplicated with shared regulon members: cspA (from cspBEG), galR (from galS), marA (from soxS or rob)

Other duplicates: appY (from gadW and others), iclR (from allR), ydeO (from gadW or appY)

Comments:

- As explained in the text, alpA, gutM, and hipB were co-transferred with nearby genes. Also, RegulonDB and ColiNet report that alpA regulates slp, but the original publication (Trempy *et al.*, 1994) gives the sequence of the regulated gene (which they term slpA), and the sequence matches that of intA (also known as b2622), rather than that of slp. IntA is separated from alpA by a single gene, so alpA is not classified as a neighbor regulator.

- AlpA, cueR, deoR, and kdgR show repeated HGT within γ-Proteobacteria.
- CueR is adjacent to its regulated gene copA in other Enterobacteria, but in *E. coli* these are separated by two other genes. This suggests that cueR was originally a neighbor regulator.
- FabR, which regulates the synthesis of unsaturated fatty acids, is adjacent to a fatty acid desaturase in Shewanella. Although that enzyme is not conserved in *E. coli*, this suggests that fabR may have originally been a neighbor regulator.
- The history of hupA is unclear. It is clearly related to hupB, together with which it forms a heterodimer known as HU, but intermediate relatives are from distant taxa. Furthermore, hupB seems to lack close paralogs in distant γ-Proteobacteria, and Shewanellas seem to have a different paralog of hupB. Hence, hupA seems to have arisen recently, perhaps by acquisition from distant organisms, and so it is classified as HGT.
- PdhR is an ancient paralog of lldR (also known as lctR), with which it shares regulation. Because this event occurred before the divergence of *E. coli* from *Shewanella*, it was classified as native.

Putative (Uncharacterized) Regulators

We examined a random sample of 20 out of 96 putative regulators (predicted regulators that are not reported to regulate any genes in RegulonDB 5.6). We verified that the putative regulators were predicted to contain DNA binding domains and that these DNA binding domains were not predicted to have other non-regulatory functions. As of May 31, 2007, EcoCyc does not report any function for these genes. A few of them are in operons with other characterized genes and hence have been given names.

Native: none

Co-transfer: b2382 (with b2383), ybcM (with ybcL), ybhD (with ybhH), ybiH (with b0795), yfeG (with eutBC), yfhA (with yfhKG) ygeV (with ygeWXYZ), yneJ (with b1525), ynfL (with ynfM)

Other HGT: b1439, b1506, b1770, yahA, yfhH, yidL, yihL, ykgD

Duplicated: yijO (from b2382), ykgK (from yqeH)

ORFan: b1685

Of these HGT genes, we classified 7 as repeated HGT within γ-Proteobacteria: b1439, yahA, yfhA, yfhH, yihL, ykgD yneJ
