# Supplementary material for: Horizontal gene transfer and the evolution of transcriptional regulation in Escherichia coli
Source: Genome Biol. 2008 Jan 7;9(1):R4. doi: 10.1186/gb-2008-9-1-r4 (PMC2395238; doi:10.1186/gb-2008-9-1-r4)
Supplement: Additional data file 2 — The figure plots the sequence conservation of regulators against the number of genes that they regulate. [file gb-2008-9-1-r4-S2.pdf]

Figure S1: Sequence conservation of regulators correlates with the number of genes that they regulate

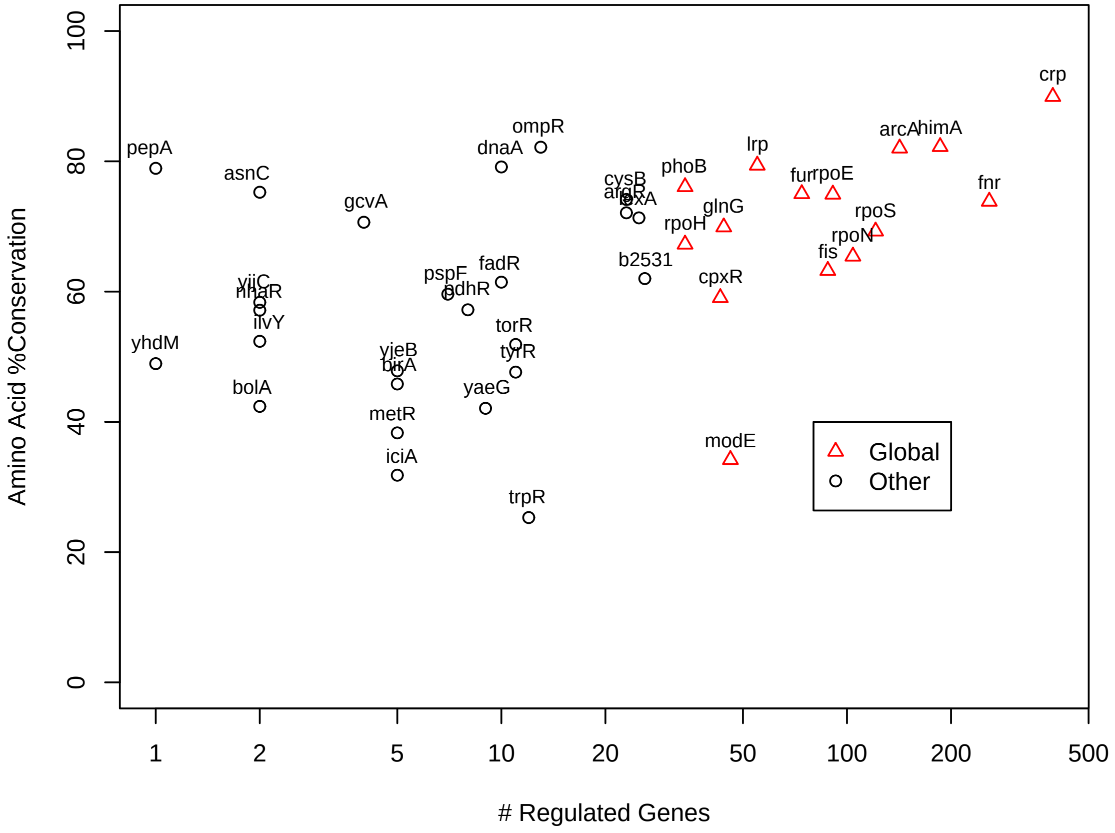

We plot the conservation (the BLAST bit score between *E. coli* and *S. oneidensis* MR-1, as a percentage of the self score), versus the number of genes that the TF regulates. Only evolutionary orthologs, as identified by PhIGs (P.S. Dehal and J.L. Boore, BMC Bioinformatics 7:201), are shown. The correlation between conservation and the number of genes regulated is statistically significant (Spearman $\rho = 0.48$, $P < 0.002$; Pearson $r = 0.49$, $P < 0.002$). Two of the most strongly conserved non-global regulators have other functions that may explain their conservation: pepA is a peptidase and a site-specific recombinase, and dnaA regulates the initiation of DNA replication.
