# Supplementary material for: Horizontal gene transfer and the evolution of transcriptional regulation in Escherichia coli
Source: Genome Biol. 2008 Jan 7;9(1):R4. doi: 10.1186/gb-2008-9-1-r4 (PMC2395238; doi:10.1186/gb-2008-9-1-r4)
Supplement: Additional data file 3 — The figure illustrates the preference for HGT between related genomes. [file gb-2008-9-1-r4-S3.pdf]

Figure S2: A preference for HGT between related genomes

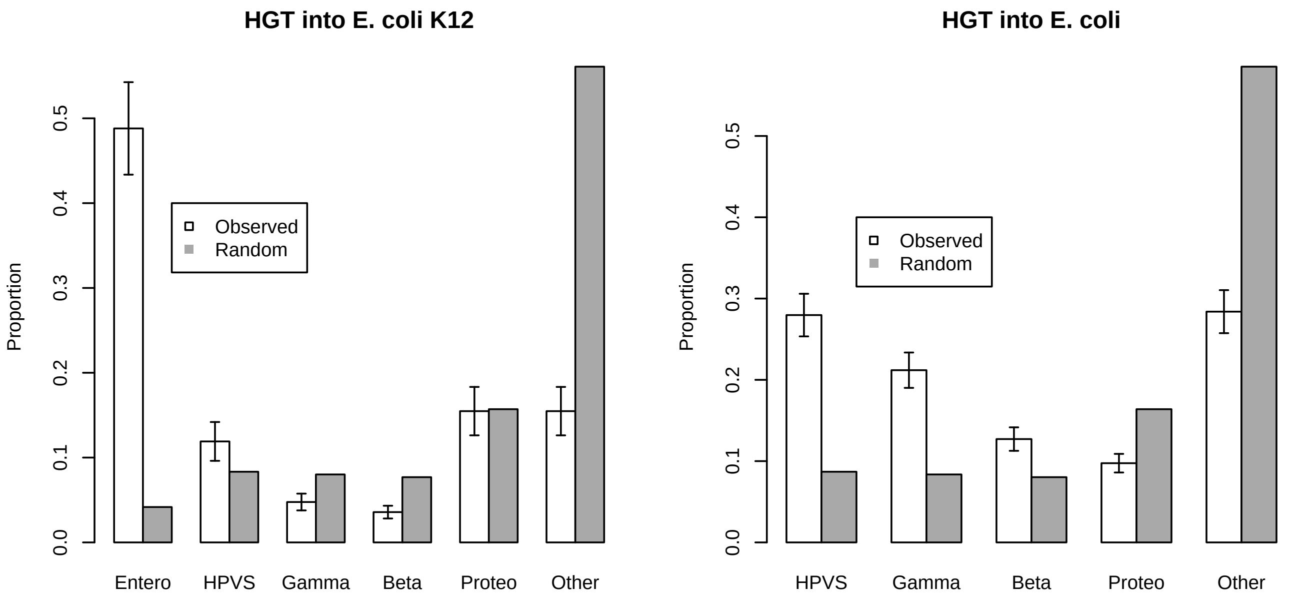

We tabulate the potential sources (as identified by best hits) of genes that were acquired by *E. coli* K12 after its divergence from other strains of *E. coli* (left panel) and the sources of genes that were acquired after the divergence of *E. coli* from *Salmonella* (right panel). We compare the distribution to that if the sources of genes were chosen at random from completely sequenced genomes. Error bars show the mean $\pm$ two standard deviations according to the binomial distribution. The most closely related groups of genomes are at the left, and the groups' names are abbreviations from Figure 9. Potential HGT events from other *E. coli* strains, from *Salmonella*, or (on the right) from other Enterobacteria are not considered because they are too close for HGT events to be identified by the automated method.
