# Supplementary material for: Horizontal gene transfer and the evolution of transcriptional regulation in Escherichia coli
Source: Genome Biol. 2008 Jan 7;9(1):R4. doi: 10.1186/gb-2008-9-1-r4 (PMC2395238; doi:10.1186/gb-2008-9-1-r4)
Supplement: Additional data file 4 — Provided is an evolutionary analysis of 30 cases of shared regulation between homologous genes from the report by Teichmann and Babu [4]. [file gb-2008-9-1-r4-S4.pdf]

Note S2: Evolutionary ages of paralogous regulatory interactions – are they conserved from a common ancestor?

We analyzed a random sample of the paralogous regulatory interactions reported by Teichmann and Babu (2004).

TF regulates two paralogous genes

Teichmann and Babu (2004) propose that if a TF regulates two paralogous genes, then this evolved by the duplication of the regulated gene together with its promoter region. We examined 10 examples at random (from “Model 1” in their supplementary material, http://www.mrc-lmb.cam.ac.uk/genomes/madanm/net_evol/ec_m1_272.txt) and classified them as follows.

Paralogous genes diverged before the regulatory interaction evolved (6):

- arcA → aceB & glcB: glcB was acquired after the divergence of *E. coli* from *Salmonella*, and glcB has close relatives in diverse bacteria including Bacilli. arcA has evolutionary orthologs only within γ-Proteobacteria (e.g. it has a paralog torR within this group). This suggests that glcB was acquired from a bacterium that did not contain arcA and hence that this regulation evolved after the divergence of aceB from glcB.
- arcA → cyoC & sdhC & sdhD: sdhC and sdhD are ancient enzymes in the TCA cycle, and hence this divergence presumably predates arcA. cyoC is also present in diverse organisms.
- crp → rhaA & yiaR: crp is unique to Proteobacteria, while rhaA and yiaR both show recent HGT with other phyla.
- fis → leuP & many other tRNAs: fis is unique to Proteobacteria, and distant relatives in other Proteobacteria have other functions (e.g., ntrC in Rhizobium). It is possible that some of these regulated tRNAs evolved by duplication (e.g., S. Giroux and R. Cedergen, J. Bacteriology 171:6446-54), but presumably most of these tRNAs diverged from each other before the Proteobacteria arose.
- himA → hycG & nuoB: himA is well-conserved within Proteobacteria, but it does not have orthologs in most other phyla (e.g., Firmicutes and Cyanobacteria, although these do contain homologous HU-like proteins). Thus, himA is ancient, but probably not older than the Proteobacteria. hycG is a recent paralog of hyfI and their closest relative is from Firmicutes (e.g., *Thermoanaerobacter tengcongensis*), so the hycG/hyfI ancestor was probably acquired from bacteria that did not have himA.

- narL → fdnI & frdC & frdD: narL is relatively recent HGT within the γ-Proteobacteria, and furthermore is a paralog of narP within γ-Proteobacteria. Only narL is reported to regulate frdABCD, while fdnGHI has sites for both as well as some narL-only sites. frdC and frdD are very distantly related (the homology is not detectable by BLAST and they are assigned to different PFams). frdCD is native to Shewanella and then has homologs in Chromobacterium and Mycobacteria, which do not contain narL/narP, which suggests that its regulation arose after it was acquired.

Operon complications (1):

- himA (IHF) → tdcE & pflB: tdcE and pflB are relatively recent paralogs, and IHF is well conserved within Proteobacteria, so this could be an ancestral relationship. However, these genes are in operons with non-paralogous genes, and neither tdcE nor pflB is the first gene in their operon, so this cannot have evolved by simply duplicating a gene together with its promoter region.

Unclear (1):

- cysB → cysH & cysM: cysH and cysM are ancient paralogs of each other and also of cysK. cysB has a paralog cbl (also known as metC) within β, γ-Proteobacteria, so it is possible that their common ancestor regulated cysH and cysM. However, more distant relatives of cysB are found in diverse bacteria and probably have diverse functions. For example, a subfamily found in Xanthomonas campestris (YP_241927) and other γ-Proteobacteria has been co-transferred with genes for leucine synthesis. Thus, we doubt whether the regulatory role of cysB/cbl is as old as the divergence of cysH from cysM.

Evolution by duplication (1):

- fliA → tarT, tap, tsr. tar/tsr are recent paralogs, and fliA is widely conserved in γ-Proteobacteria.

Other (1):

- purR → gcvP: The only paralog of gcvP we identified was rtcB, which is not regulated by purR, so we do not know why this was included in Teichmann & Babu's analysis.

Paralogous TFs regulate the same gene

Teichmann and Babu (2004) propose that if paralogous TFs regulate the same gene, then this evolved by the duplication of the TF. We examined 10 examples at random (from "Model 2" in their supplementary material, http://www.mrc-lmb.cam.ac.uk/genomes/madanm/net_evol/ec_m2_128.txt) and classified them as follows.

TF duplication predates acquisition of regulated gene (6):

- arcA & dcuR → dctA: As discussed above, arcA is orthologous within γ-Proteobacteria. dcuR, which is also known as yjdG, is present in Enterobacteria but not in more distantly-related γ-Proteobacteria and seems to have been acquired by HGT, perhaps from Firmicutes.
- arcA & narL → nuoL: nuoL is in Enterobacteria but not in other relatives and seems to have been acquired by HGT. Both arcA and narL have older origins within the γ-Proteobacteria.
- arcA & narL → nuoN: As with nuoL, nuoN is in Enterobacteria but not in most other relatives.
- cbl & cysB → tauD: cysB and cbl are paralogs within the β, γ-Proteobacteria, while tauD is present in Enterobacteria such as Yersinia but not in more distant γ-Proteobacteria.
- crp & fnr → ansB: ansB was acquired by HGT, probably after the divergence of *E. coli* from *Vibrio* species, and has close homologs in other phyla, while crp and fnr are ancient native genes within Proteobacteria.
- lysR & tdcA → tdcA: lysR has a complex history of HGT, as does tdcA, and these genes are distantly related.

Unclear (4):

- crp & fnr → sucA: Both regulators and the regulated gene are ancient native genes within Proteobacteria, so it is hard to determine if the duplication predates the regulation or not.
- crp & fnr → sucB: Both regulators and the regulated gene are ancient native genes within Proteobacteria, so it is hard to determine if the duplication predates the regulation or not.
- crp & fnr → tdcA: The regulation of tdcA by fnr seems to be indirect (Chattopadhyay et al., J. Bacteriol. 179:4868-73). In any case, crp and fnr are ancient paralogs and are both highly conserved within γ-Proteobacteria. tdcA is a recent paralog of ydaK and has a complex history of HGT before that. Because many of these homologs are in β, γ-Proteobacteria, we cannot rule out the possibility that tdcA was regulated by crp and fnr before the acquisition.
- crp & fnr → tdcG: tdcG is in the same operon as tdcA and has the same history.

Paralogous TFs regulate paralogous genes

Teichmann and Babu (2004) propose that if paralogous TFs regulate paralogous genes, then this evolved by the duplication of both the TF and the regulated genes. We examined 10 examples at random (taken from "Model 3" in their supplementary material, http://www.mrc-lmb.cam.ac.uk/genomes/madanm/net_evol/ec_m3_74.txt) and classified them as follows.

Autoregulation of distantly-related TFs (4):

- arsR → arsR & marR → marR.
- betI → betI & uidR → uidR.
- asnC → asnC & lrp → lrp.
- galS → galS & idnR → idnR.

(Because auto-regulation is common for all types of transcription factors, it is not surprising that distantly-related pairs of TFs are found in which both members of the pair regulate their own transcription. Hence, there is no reason to expect that this reflects conserved regulation from a common ancestor.)

Unclear (2):

- evgA → ompC & ompR → fadL, ompC, ompF & phoB → phoE : These two-component systems have different functions and have closer paralogs: evgA has closer paralogs bglJ, dctR, and rcsA; phoB has closer paralogs baeR and creB; ompR has closer paralogs cpxR and torR. This suggests that the regulatory cross-talk between these two-component systems arose after the duplication events.
- fur → fepC & fhuC & zur → znuC. (zur is also known as yjbK and znuC is also known as yebM.) zur and fur are ancient paralogs. fepC, znuC, and fhuC all show evidence for recent HGT. However, because zur is adjacent to znuC in some distant bacteria, it is possible that the two have co-evolved and have been co-transferred since the divergence.

Other (4):

- gcvA → gcvP: We did not find regulatory relationships between paralogs for gcvA (cbl, cynR, cysB, dsdC, hcaR, ilvY, lysR, metR, nac, nhaR, oxyR, tdcA, xapR) and gcvP (rtcB), so we do not know why this was included in Teichmann & Babu's analysis.

- marR → nfo: We did not find regulatory relationships between paralogs for marR (arsR,gatR_2) and nfo (rhaA,uxuA,xylA,yiaR), so we do not know why this was included in Teichmann & Babu's analysis.
- metR → glyA: We did not find regulatory relationships between paralogs for metR (cbl, cynR, cysB, dsdC, gcvA, hcaR, ilvY, lysR, nac, nhaR, oxyR, tdcA, xapR) and glyA (argD, bioA, bioF, metC, kbl, malY, tnaA, tyrB), so we do not know why this was included in Teichmann & Babu's analysis.
- yiaJ → yiaQ: We did not find any regulatory relationships between paralogs for yiaJ (iclR,mhpR) and yiaQ (trpC), so we do not know why this was included in Teichmann & Babu's analysis.
