# Supplementary material for: Horizontal gene transfer and the evolution of transcriptional regulation in Escherichia coli
Source: Genome Biol. 2008 Jan 7;9(1):R4. doi: 10.1186/gb-2008-9-1-r4 (PMC2395238; doi:10.1186/gb-2008-9-1-r4)
Supplement: Additional data file 5 — Provides an evolutionary analysis of each case of shared regulation between 'close' paralogs. [file gb-2008-9-1-r4-S5.pdf]

Note S3: Evolutionary ages of regulatory interactions shared by close paralogs

We examined all regulatory interactions in RegulonDB 5.6 that are shared between "close" paralogs (defined by a BLAST bit-score above 30% of self-score). We removed purely autoregulatory interactions but not other regulation in the same operon: e.g., if operon A-B is regulated by A, then we still consider the regulatory interaction between A and B. Although we exclude autoregulation from consideration, this does not mean that autoregulation cannot evolve independently (autoregulation seems to be poorly conserved, see Results).

After excluding autoregulation, we found 212 cases where paralogous TFs regulated the same operon, 290 cases where paralogous genes were regulated by the same TF, and 54 cases where paralogous TFs regulated paralogous genes.

Paralogous TFs regulate the same operon

By eliminating mirror symmetry (e.g., if TF1 and TF2 both regulate the same gene, then we can ignore the redudant observation that TF2 and TF1 both regulate that gene), 212 cases were reduced to 106. These cases further reduced to 35 operons, which are listed below. Note that autoregulated genes (in italics) are included in the operons below, but are not included in the count of 106 cases or genes.

Convergent evolution (10 regulated operons, 32 regulated genes):

**TF1**	**TF2**	**Regulated Operon**	**Cases**
Regulated genes acquired after the duplication; TFs bind different sites			
arcA	torR	gadAX (arcA's site is predicted to be far upstream, J Biol Chem. 280:15084–96)	2
baeR	cpxR	acrD	1
baeR	cpxR	mdtABCD-baeS*R*	5
cbl	cysB	ssuEADCB	5
Regulated genes acquired after the duplication; sites not known			
cbl	cysB	tauABCD	4
narL	narP	fdhF	1
Regulated genes acquired after the duplication, but TFs bind the same site			
gntR	idnR	idnDOT*R*	3
narL	narP	fdnGHI	3
narL	narP	hcp-hcr	2
narL	narP	hyaABCDEF	6

Unclear (10 regulated operons, 32 regulated genes):

TF1	TF2	Regulated Operon	Cases
Relative ages unclear, and the TFs bind the same site			
cpxR	ompR	csgDEFG	4
cpxR	ompR	ompC	1
cpxR	ompR	ompF	1
fhlA	hyfR	hyfABCDEFGHIJ*R*-focB	11
gntR	idnR	idnK	1
marA	soxS	acrAB	2
marA	soxS	marR*A*B	2
narL	narP	norVW	2
narL	narP	nrfABCDEFG	7
TF duplication is younger than the regulated gene, but the sites are different			
marA	soxS	fumC	1

Evolution by duplication (15 operons, 42 regulated genes):

TF1	TF2	Regulated Operon	Cases
TF duplication is probably younger than the regulated gene, and the site is shared			
exuR	uxuR	uxuAB	2
galR	galS	galETKM	4
galR	galS	galP	1
galR	galS	mglBAC	3
gntR	idnR	gntKU	2
marA	soxS	fpr	1
marA	soxS	inaA	1
marA	soxS	nfo	1
marA	soxS	poxB	1
marA	soxS	pqiAB	2
marA	soxS	sodA	1
marA	soxS	zwf	1
mlc	nagC	manXYZ	3
narL	narP	napFDAGHBC-ccmABCDEFGH	15
narL	narP	nirBDC-cysG	4

Comments:

ompC and ompF have a complex history of additional duplication events after the original divergence. Some of the extra paralogs have similar regulation, but none of them are regulated by both cpxR and ompR. Nevertheless, the regulation of these genes could have been conserved, while that of the other paralogs diverged.

The regulation of the mdtA promoter by baeR and cpxR seems to have evolved independently – they are reported to bind to distinct upstream sites, and baeSR and mdtABCD have been acquired by HGT after the divergence of Enterobacteria. Thus, even though baeR has probably coevolved

with its histidine kinase baeS in an operon for a long time (perhaps even since baeSR diverged from cpxRA), the regulation of baeSR by cpxR appears to have evolved recently. There is also an internal promoter upstream of baeSR, which has not, to our knowledge, been characterized. If there is any ancient conserved autoregulation of baeSR by baeR, it probably acts there, not at mdtA's promoter.

In RegulonDB, poxB is listed as being in an operon with downstream genes ltaE and ybjT, but only poxB is listed as being regulated by marA or soxS. (This appears to be an omission in RegulonDB.) Similarly, gntKU are listed as being in an operon with the upstream gene gntR, but only gntKU are regulated by gntR and idnR, because of an internal promoter upstream of gntK.

Paralogous Genes Are Regulated by the Same TF

By eliminating mirror symmetry, 290 paralogous gene-TF interactions reduced to 145 cases. In only 62 of these cases were the first two genes in the operon close homologs, so that the operon structures were consistent with evolution by duplication. We classified those and list them below. Usually, not all the genes in the operons are paralogs – the paralogs are in bold. We also examined some of the cases where the operon structures were not consistent with evolution by duplication, and confirmed that these usually involved ancient duplications or movement of a gene to a preexisting operon (data not shown).

Evolution by duplication (17 duplication events accounting for 38 cases):

**Operon**	**Operon**	**TF(s)**	**Cases**
**araE**	**galP**	crp	1
**argF**	**argI**	argR	1
cyd**AB**	app**CB**A	arcA	2
dms**ABC**	ynf**EFGH**-dmsD	fnr	4
**fimB**	**fimE**	hns	1
**fldA**-fur	**fldB**	soxS	1
gad**BC**	gad**AX**	gadE, crp, hns, rpoS, gadW, gadX	6
gnt**K**U	**idnK**	crp, gntR, idnR	3
liv**K**HMGF	**livJ**	lrp	1
**nmpC**	**ompC**	himA, ompR	2
**nmpC**	**ompF**	crp, himA, ompR	3
**ompF**	**ompC**	cpxR, envY, himA, lrp, ompR	5
**tar**-tap-cheRBYZ	tar-**tap**-cheRBYZ	fliA, fnr	2
**tar-tap**-cheRBYZ	**tsr**	fliA	2
**trg**	**tar-tap**-cheRBYZ	fliA	2
**trg**	**tsr**	fliA	1
ynf**E**FGH-dmsD	ynfE**F**GH-dmsD	fnr	1

Unclear cases involving long-diverged genes – 7 putative duplication events accounting for 13 cases:

**Operon**	**Operon**	**TF(s)**	**Cases**
**argT**-hisJQMP	argT-his**J**QMP	glnG, rpoN	2
**astC**ADBE	**argD**	argR	1
**gapA**	**epd**-pgk-fbaA	crp	1
ibp**A**B	ibpA**B**	rpoH	1
mdtA**B**CD-baeSR	mdtAB**C**D-baeSR	baeR, cpxR	2
oppABC**D**F	oppABCD**F**	arcA, lrp, modE	3
**sodB**	**sodA**	crp, fur, himA	3

Covergent evolution, where one of the regulated genes was acquired after the duplication – 7 putative duplication events accounting for 11 cases:

**Operon**	**Operon**	**TF(s)**	**Cases**
**aroG**	**aroF**-tyrA	tyrR	1
dsd**X**A	**gntT**	crp	1
**gntT**	**gntP**	crp	1
gudP**X**D	gudPX**D**	yaeG	1
gud**P**XD	gar**P**LRK-rnpB	yaeG	1
**hupB**	**hupA**	crp, fis	2
hya**AB**CDEF	hyb**O**AB**C**DEFG	arcA, narL	4

Comments:

mdtB/mdtC and oppD/oppF might have arisen from an ancient tandem duplication event, followed by evolution of new regulation for the operon in the *E. coli* lineage.

The proximity of gudX and gudD suggests a tandem duplication, but the phylogenetic tree implies otherwise – gudD is present only in Enterobacteria, and it branches more deeply than gudY from other lineages.

Paralogous Genes Are Regulated by Paralogous TFs

By eliminating mirror symmetry, 54 cases were reduced to 27, which are listed below:

Duplication of both TF and regulated gene(s) – 4 cases:

**TF**$_1$	**Operon**$_1$	**TF**$_2$	**Operon**$_2$	**Cases**
gatR	gatY**Z**ABCD	agaR	aga**Z**VWA	1
gatR	gat**Y**ZABCD	agaR	agaS**Y**BCDI	1
gntR	**idnK**	idnR	gnt**K**U	1
gntR	gnt**K**U	idnR	**idnK**	1

Convergent evolution, with complex HGT of regulated genes – two putative duplication events, 8 cases:

**TF_1**	**$Operon_1$**	**TF_2**	**$Operon_2$**	**Cases**
narL	hyb**O**AB**C**DEFG	narP	hya**AB**CDEF	2
hyfR	hyf**A**B**C**DEF**GHIJ**R-focB	fhlA	hyc**A**B**C**D**EFGH**I	6

Convergent evolution – TF duplication precedes that of regulated genes – 15 cases:

**TF_1**	**$Operon_1$**	**TF_2**	**$Operon_2$**	**Cases**
baeR	mdtA**B**CD-baeSR	cpxR	mdtAB**C**D-baeSR	1
baeR	mdtAB**C**D-baeSR	cpxR	mdtA**B**CD-baeSR	1
cpxR	**ompC**	ompR	**nmpC**	1
cpxR	**ompC**	ompR	**ompF**	1
cpxR	**ompF**	ompR	**nmpC**	1
cpxR	**ompF**	ompR	**ompC**	1
gntR	**gntT**	idnR	gnt**KU**	1
gntR	idnDO**T**R	idnR	gnt**KU**	1
gntR	**gntT**	idnR	idnDO**T**R	1
gntR	gnt**KU**	idnR	idnDO**TR**	1
phoB	**phoE**	ompR	**nmpC**	1
phoB	**phoE**	ompR	**ompC**	1
phoB	**phoE**	ompR	**ompF**	1
phoB	**phoE**	cpxR	**ompC**	1
phoB	**phoE**	cpxR	**ompF**	1

Comments: the similar regulation of ompC, ompF, and nmpC probably reflects duplication of the genes (but not of the TFs). gatR and agaR appear to have co-evolved with their regulated operons, despite more recent HGT events and changes in operon structure.
